# Human cortical spheroids with a high diversity of innately developing brain cell types

**DOI:** 10.1186/s13287-023-03261-3

**Published:** 2023-03-23

**Authors:** Kim M. A. De Kleijn, Wieteke A. Zuure, Kirsten R. Straasheijm, Marijn B. Martens, M. Cristina Avramut, Roman I. Koning, Gerard J. M. Martens

**Affiliations:** 1 Department of Molecular Animal Physiology, Donders Institute for Brain, Cognition and Behavior, Centre for Neuroscience, Faculty of Science, Radboud University, 6525GA Nijmegen, The Netherlands; 2 NeuroDrug Research Ltd, 6525ED Nijmegen, The Netherlands; 3 Department of Cell and Chemical Biology, Leiden University Medical Center, 2300RC Leiden, The Netherlands

**Keywords:** Brain organoid, Brain spheroid, Neuroectoderm, Mesoderm, Glial cells, Endothelial cells, Embryonic stem cell, Neuronal cells

## Abstract

**Background:**

Three-dimensional (3D) human brain spheroids are instrumental to study central nervous system (CNS) development and (dys)function. Yet, in current brain spheroid models the limited variety of cell types hampers an integrated exploration of CNS (disease) mechanisms.

**Methods:**

Here we report a 5-month culture protocol that reproducibly generates H9 embryonic stem cell-derived human cortical spheroids (hCSs) with a large cell-type variety.

**Results:**

We established the presence of not only neuroectoderm-derived neural progenitor populations, mature excitatory and inhibitory neurons, astrocytes and oligodendrocyte (precursor) cells, but also mesoderm-derived microglia and endothelial cell populations in the hCSs via RNA-sequencing, qPCR, immunocytochemistry and transmission electron microscopy. Transcriptomic analysis revealed resemblance between the 5-months-old hCSs and dorsal frontal rather than inferior regions of human fetal brains of 19–26 weeks of gestational age. Pro-inflammatory stimulation of the generated hCSs induced a neuroinflammatory response, offering a proof-of-principle of the applicability of the spheroids.

**Conclusions:**

Our protocol provides a 3D human brain cell model containing a wide variety of innately developing neuroectoderm- as well as mesoderm-derived cell types, furnishing a versatile platform for comprehensive examination of intercellular CNS communication and neurological disease mechanisms.

**Supplementary Information:**

The online version contains supplementary material available at 10.1186/s13287-023-03261-3.

## Introduction

Three-dimensional (3D) stem-cell-derived human cortical spheroids (hCSs) are valuable to explore human brain development, function and disease, with early and mature hCSs modelling neurodevelopmental and neurodegenerative diseases, respectively [1]. Since the first creation of hCSs in a guided [2] or non-guided [3] manner, their complexity and cellular diversity has increased such that now the major neuroectoderm-derived cell types can be produced in 3D [4–6]. In silico analysis of eight distinct hCSs [7], each produced with a different (guided or non-guided) culture protocol, showed that the highest degree of diversity of innately grown brain cells was generated with a non-guided protocol [8]. The induction of neurons, astrocytes and myelinating oligodendrocytes has been reported in spheroids derived from induced pluripotent stem cells (iPSCs) or embryonic stem cells (ESCs) [9–13]. Still, most current hCS protocols do not lead to innately developing microglia, the immune cells of the central nervous system (CNS), and vascular endothelial cells that may mimic the blood–brain-barrier (BBB). In an attempt to reach a higher level of complexity, microglial cells differentiated from iPSCs or a microglial cell line have been incorporated into iPSC-derived hCSs already containing neuroectoderm-derived neurons, astrocytes and oligodendrocytes [14–18]. Such efforts have been undertaken since an innate development of microglial cells in hCSs is not immediately obvious as microglia (and also endothelial cells) do not originate from neuroectoderm, but from the mesodermal epithelium of the embryonic yolk sac [19]. Mature and inducible microglia have been found to innately grow in iPSC-derived hCSs containing neuronal and astrocyte populations, although in these spheroids the presence of other mesoderm-cell types and oligodendrocyte populations was not reported [20]. Furthermore, hCSs containing endothelial cells have been generated via co-culturing hCSs with iPSC-derived endothelial cells [21] or with human umbilical vascular endothelial cells [22]. Others have co-cultured human brain organoids and blood vessel organoids [23–25] or genetically engineered ESCs to create vascular endothelial cells in human cortical organoids [26]. However, in these neuroectoderm-endothelial hybrid spheroids the presence of innately grown microglia cells has not been reported. Assembly of 3D hCSs from various 2D iPSC-derived hCS cultures did result in a model comprising all major CNS cell types, including microglia and endothelial cells [27]. Nevertheless, such an assembly method does not allow for analysis of the stepwise innate development of the various cell types into a complex system. Thus, there is a shortcoming within the field regarding the availability of a guided iPSC- or ESC-derived hCS model which contains not only innately grown neurons, astrocytes and oligodendrocytes, but in the same spheroid also innately grown (and not co-cultured or fused) microglia and endothelial cells.

For the present study, it is important to note that the lack of dual Small Mothers Against Decapentaplegic (SMAD) inhibition enables mesoderm germ layer development in iPSC-derived hCSs [8, 20] and that Bone morphogenetic protein 4 (BMP4), acting upstream of SMAD, is involved in mesoderm development [28, 29]. We therefore hypothesized that mild dual-SMAD inhibition (via reduced BMP4 inhibition) during early cortical patterning may allow for the development of mesoderm-derived cell types in neuroectoderm-guided hCSs. We find that a reduced concentration of dorsomorphin (DM), a BMP4 signaling inhibitor, indeed permits the innate development of ESC-derived hCSs containing not only neurons, astrocytes and oligodendrocytes, but also various microglial subtypes and endothelial lineage cells. The cell-type composition of our 150-days-old hCSs is remarkably similar to those of dorsal cortical, rather than more inferior, regions of the midgestational (weeks 19–26) human fetal brain. Analysis of the response of our hCSs to a pro-inflammatory stimulus shows that the generated spheroids are helpful to gain pathophysiological insight into neuroinflammatory processes.

## Materials and methods

### H9 ESC cultures

Plating of H9 ESCs (WA09; WiCell, passage number 24) was performed in E8 medium with Revitacell (Thermo Fisher, A26445-01). H9 ESCs were grown feeder dependent on mouse embryonic fibroblasts (Thermo Fisher, A34958) for 5 passages and were then transferred to feeder-independent culturing. H9 ESCs were grown on surfaces coated with Matrigel (Corning, 354,277) diluted 1:60 in Dulbecco's phosphate-buffered saline (DPBS) (Gibco, 14190–094) in E8 medium (Gibco, A15169-01) supplemented with Penicillin–Streptomycin (pen-strep, 1:100, Thermo Fisher, 15140–122). On average, H9 ESCs were passaged twice per week at a 1:25 splitting ratio. Cells were dissociated by a 5-min incubation with Accutase (Invitrogen, 00–4555-56), centrifuged at 1000 rpm for 5 min in DPBS and resuspended in room-temperature E8 medium. Mycoplasma tests (Lonza, LT07-118) were performed 5 times per year to ensure the mycoplasma-free status of our cultures. Daily visual inspections were performed to ensure the undifferentiated state of the stem-cell cultures, which was confirmed by cell-marker mRNA expression analysis over several passages (Additional file 1: Fig. S1). Any differentiated cells were marked with a cell-culture marker and the area was removed by micropipette suction.

### 3D hCS formation

As a first step towards 3D hCS formation, intact H9 ESC-colonies were detached with ReLESR (STEMcell technologies, 05,872), a small volume of cell suspension was dissociated into single cells and counted, and low-adherence V-bottom 96-wells (S-Bio, #MS-9096VZ) were seeded with 1.25*10^4^ cells per well in 190 μl Spheroid Starter Medium (SSM: DMEM-F12 (Gibco, 31331–028) with 20% knock-out serum (Gibco, 10828–028), 1% non-essential amino acids (Gibco, 11140,050), 1% pen-strep and 0.1% 2-mercaptoethanol (Thermo Fisher, 31350–010)) supplemented with 10 μM SB-431542 (Sigma-Aldrich, S4317), 1 μM DM (Sigma-Aldrich, P5499) and 2 μM ROCK inhibitor thiazovivin (Sigma-Aldrich, SML1045). SSM (190 μl) supplemented with 10 μM SB-431542 and 1 μM DM and was refreshed daily for seven days. Subsequently, the medium was replaced by Neurobasal-A spheroid medium (Thermo Fisher, 10888–022) with 2% B27 supplement minus vitamin A (Gibco; 12587–010), 1% GlutaMAX (Gibco, 35050–061) and 1% pen-strep (NSMnogel) supplemented with 25 μg/ml FGF2 (Sigma-Aldrich, F0291) and 10 μg/ml EGF (Gibco, PHG0311L), and was refreshed daily until day (d) 17 (d17) and half of the medium refreshed every other day from d17 until d25. Then, spheroids were transferred to low-attachment 6 cm dishes (~ 24 spheroids in 6 ml per dish) with a cut-open P1000 tip in NSM (NSMnogel containing 15 μg/ml Geltrex (Thermo Fisher, A1413302)) supplemented with the neural differentiation factors 25 μg/ml FGF2 and 10 μg/ml EGF. Half of the medium was refreshed with NSM medium supplemented with 20 ng/ml of the neuronal differentiation factors NT-3 (Sigma-Aldrich, SRP3128) and BDNF (Sigma-Aldrich, SRP3014) every other day from d27 until d43. Between d43 and d51 half of the NSM was refreshed every other day with NSM (no supplements). From d51 until d59, half of the medium was refreshed every other day with NSM supplemented with the oligodendrocyte precursor cell (OPC) differentiation factors 10 ng/ml PDGF-AA (Sigma-Aldrich; H8291) and 10 ng/ml IGF1 (R&D systems; 291-G1-200). From d61 until d73, half of the medium was refreshed every other day with NSM medium containing the pro-myelinating factor 4 μM ketoconazole (Sigma-Aldrich, K1003). Finally, from d73 onwards, half of the medium was refreshed every other day with NSM medium (no supplements) until the spheroids were harvested. This protocol was designed in order to allow for mesoderm development alongside neuroectoderm development in our 3D spheroids and was adapted from Madhavan et al. (2018), with a tenfold reduction in the concentration of the BMP4 signaling inhibitor DM being the major modification. Furthermore, the modifications included less-frequent medium changes during the progenitor differentiation stage (d17 to d25) to reduce the propensity towards neuroectoderm-only differentiation and the use of a different ROCK inhibitor. During the entire growth period and all experiments, involving the culturing of 13 independent hCS batches, hCSs were kept at 37 °C and 5% CO2. Batch-1 (hCSs-1) was used for quantitative polymerase chain reaction (qPCR) experiments to compare hCSs with H9 cells. Batch-2 (hCSs-2), batch-3 (with two technical replicates, hCSs-3_1 and hCSs-3_2), batch-4 (with two technical replicates, hCSs-4_1 and hCSs-4_2), batch-5 (hCSs-5), batch-6 (hCSs-6) and batch-7 (hCSs-7) were used for the time-point qPCR experiments. For six batches, the number of spheroids was not sufficient to cover all time points in the time-point qPCR experiments with these batches. Thus, we did not perform a time-course comparison, but rather a comparison of independently grown batches at several time points. We acknowledge that, in addition to the ESC-line that was used as a negative control, neural stem cells or differentiated ESCs could have served as a positive control in our qPCR experiments. We did test the primers for the well-validated and extensively studied markers used to identify the various ESC-derived cell types in a positive control tissue (human M1 cortex). Confocal imaging was performed on batch-2 hCSs. Transmission electron microscopy (TEM) imaging was performed on hCSs-2 and hCSs-13. For the Purinergic Receptor P2Y12 (P2RY12) heterogeneity experiments, hCss-1, hCSs-7, hCSs-8, hCSs-9 and hCSs-10 were used. hCSs-9 was also used for the H9 starting-cell-density experiment, hCSs-11 for the dual SMAD-omission experiment and hCSs-2 for the BMP4-modulation experiment. For comparisons between our hCSs and hCSs generated with other protocols or human fetal brain, we used hCS-batches hCSs-1 and hCSs-12.

### Pro-inflammatory stimulation of hCSs

Around d150, hCSs were transferred to low-attachment 6-well plates (Corning) and incubated with or without 100 ng/ml Lipopolysaccharide (LPS) (Sigma-Aldrich, L4391-1MG, dissolved in PBS, 2 ml total NSM per 6-well) for 24 h. Subsequently, LPS was washed off with DPBS and hCSs were incubated with or without 5 ng/ml Tumor necrosis factor alpha (TNFα) (Sigma-Aldrich, T0157-10UG, dissolved in PBS) and 5 ng/ml Interleukin-1 beta (IL-1β) (Sigma-Aldrich, SRP3083-10UG, dissolved in PBS) for 24 h. Spheroids were then harvested and processed as described below.

### RNA isolation, cDNA synthesis and qPCR analysis

H9 ESCs were lysed in TRIzol, and the RNA was extracted with chloroform, and precipitated with 100% 2-propanol and glycogen. Single hCSs were transferred with a cut-open P1000 tip to 400 μl TRIzol reagent each (Thermo Fisher, 15,596,026), snap frozen in liquid nitrogen and stored at -20 °C. For cell lysis, hCSs were incubated at room temperature in TRIzol for 5 min, then broken into smaller pieces with a P200 tip and incubated for 30 min on ice. RNA was extracted with chloroform, and precipitated with 100% 2-propanol and glycogen. H9 ESC and hCS RNAs were washed with ice-cold 75% ethanol and dissolved in MilliQ. Prior to cDNA synthesis, the RNA was treated with DNase. cDNA synthesis was performed with the Revert Aid H-minus first-strand cDNA synthesis kit (Thermo Scientific). We performed qPCR reactions with SensiFAST™ Probe No-ROX (Bioline; BIO-86005) using 1:15 diluted cDNA (for primer sequences, see Additional file 14: Table S1) in a Corbett Life Sciences Rotor-Gene 6000. The cDNA was amplified for 50 cycles of 95 °C (5 s), 60 °C (10 s) and 72 °C (15 s), and melt-curve analysis was performed to confirm correct amplicon size. Gene-of-interest values were normalized with a factor based on the two most stably expressed housekeeping genes out of Glyceraldehyde 3-phosphate dehydrogenase (GAPDH), 14–3-3 protein zeta/delta (YWHAZ), Eukaryotic Translation Initiation Factor 4A2 (EIF4A2) or Peptidylprolyl Isomerase A (PPIA), with low M-value (< 0.5) as calculated by GeNorm. Normalized mRNA expression (Q-value) was calculated for each sample as follows [30]:

1 $$Q_{x} = \mu_{{{\mathrm{eff}}}}^{{\left( {ct_{\min } - ct_{x} } \right)}}$$

In which $\mu_{{{\mathrm{eff}}}}$ is the average amplification efficiency of all samples amplified with a certain primer pair, $ct_{\min }$ is the minimum ct value of these samples and $ct_{x}$ is the ct value of the sample for which the $Q_{x}$-value is calculated. This formula takes into account the mean amplification efficiency for one primer pair and scales all values between 0 and 1 for relative comparisons.

### Immunocytochemistry (ICC)

For ICC, hCSs were transferred with a cut-open P1000 tip, washed once with PBS and then fixed in 4% paraformaldehyde (PFA in phosphate buffer) for 45 min at room temperature. Spheroids were then washed two times with PBS, incubated for 2 hours (2h) in 15% sucrose (in PBS) at 4 °C and then overnight at 4 °C in 30% sucrose (in PBS). We embedded spheroids in O.C.T. Compound (VWR; 361603E) and prepared 5 μm sections on SuperFrost PLUS slides (VWR; J1800AMNZ), which were frozen at -20 °C. After thawing, sections were equilibrated in PBS for 5 min and, depending on the antibody, blocked in blocking buffer (5% goat/donkey/horse serum, 1% Bovine serum albumin (BSA), 1% Glycine, 0.1% D-lysine, in PBS) with or without 0.4% Triton-X for 30 min at room temperature. Antibodies were diluted in either PBS or PBS-Tween (PBS-T) 0.1%; see Additional file 15: Table S2 for the list of antibodies used. Primary antibodies were incubated overnight at 4 °C and secondary antibodies (all diluted 1:500) for 2 h at room temperature. Depending on the antibody, washes were performed with either PBS or PBS-T 0.1%. To test for any background staining, primary antibodies were used to stain hCSs at a culture time point at which no differentiated cell types and thus no antigen expression was expected (d17), and except for the Paired box protein 6 (PAX6) antibody no signal and thus no nonspecific binding was indeed observed. In case of the PAX6 antibody, staining in the cytoplasm was observed, which was considered nonspecific and these cells were not counted as PAX6-positive cells since the transcription factor PAX6 is expressed in the nucleus. We imaged the sections using an EVOS FL Auto 2 microscope (Thermo Fisher) with a 60X Olympus oil objective (Thermo Fisher; 1.42NA/0.15WD) with the EVOS FL auto 2 acquisition software or with a confocal LSM900 Zeiss microscope with a 63X objective (Oil DIC M27; 1.4NA), a step-size of 1.4 (0.23 μm) and a 0.5-μm focal plane, with PMT detectors and applying the ZEN (version 3.1) acquisition software. Total-field or average cell-signal intensities were quantified with the Cell Viability package of Cellomics Studio Software (Thermo Fisher). Per spheroid, all fields of a section were quantified. For the analysis of spheroid sizes at different culture time points, brightfield images were taken with a 4X objective, and circumference was calculated in ImageJ and converted from pixels to mm.

### TEM

For morphological analysis, hCSs were transferred with a cut-open P1000 tip, the medium was removed and spheroids were washed twice with 0.1 M sodium-cacodylate, pH 7.4 (Sigma-Aldrich) or the Pelco BioWave Pro + system (Ted Pella, Inc.) was used. Samples were then fixed for 1 h at room temperature with 2.5% glutaraldehyde (Electron Microscopy Sciences; 16,210)/2% PFA (Electron Microscopy Sciences; 157–8) in 0.1 M sodium-cacodylate. Subsequently, spheroids were rinsed twice with 0.1 M sodium-cacodylate and fixed for 1 h on ice with 1% osmium tetroxide (Electron Microscopy Sciences; 19,152) in 0.1 M sodium-cacodylate buffer. Probes were further rinsed twice with 0.1 M sodium-cacodylate buffer and twice with MilliQ, and then en bloc stained with 1% uranyl acetate in MilliQ for 1 h at room temperature. Spheroids were subsequently washed twice with MilliQ and twice with 70% ethanol, followed by dehydration in 70% ethanol overnight, 80% ethanol (10 min), 90% ethanol (10 min), and 100% absolute ethanol (twice 15 min; once 30 min). Spheroids were then infiltrated with mixtures of ethanol, and 25%, 50% and 75% Epon LX-112 (Ladd Research; 21,210) (30 min for each step), followed by pure Epon for 90 min at room temperature and 30 min at 60 °C. Specimens were then embedded in pure Epon and polymerized for 48 h at 60 °C. Ultrathin Sects. (100 nm) were collected onto copper slot grids (Storck Veco; OD841130) covered with a formvar film and a thin carbon layer, and then stained with an aqueous solution of 7% uranyl acetate for 20 min, followed by Reynold’s lead citrate for 10 min. Samples were imaged at an acceleration voltage of 120 kV using a FEI Tecnai 12 (BioTWIN) TEM equipped with a 4 k Eagle CCD camera (FEI, Eindhoven, The Netherlands). Large virtual slides (image mosaics) containing hundreds or even thousands of images were acquired using automated data acquisition and stitching software [31]. Images were captured at 6800× magnification, with binning 2, corresponding to a 3.25-nm pixel size at the specimen level. Open-source Aperio ImageScope software (Leica Biosystems) was used for the visualization of the virtual slides. The advantage of large virtual slides is that they provide an overview of large spheroid parts while maintaining the possibility to zoom in to high detail and allow morphological analysis at high resolution.

### RNA-sequencing (RNA-seq) analysis

Bulk RNA-seq analysis of total RNA samples (RNA integrity number (RIN) > 6.8) was performed on a DNBseq platform with read depth of 30,000 reads, clean reads were mapped to reference genome h38 using HISAT2 (v2.0.4), and 18,028 (batch-1) and 17,969 (batch-12) genes were identified (BGI Genomics, Shenzhen, China). Additional files 9 and 10 give the Fragments Per Kilo base of transcript per Million mapped reads (FPKM) values per batch. The raw data of batch-1 and batch-12 are also available under GEO accession number GSE200779 (encoded “0h” and “0h_2”, respectively).

### Comparative analysis of cell-type compositions

First, brain cell-type-specific markers were selected on the basis of extensive literature searches (for the full list of markers examined, see Additional file 11), and their upregulation in single cell-type clusters derived from a human fetal brain single-cell RNA sequencing dataset [32] (a marker was considered upregulated when the median fold-change (FC) in expression over other cell-type clusters was > 3.5, with typical FC-values being > 10; Additional file 16: Table S3). The selected markers were used for the comparative analysis of the mRNA expression profiles of two of our hCS batches (batch-1 and batch-12), and the profiles of our hCSs and the hCSs generated by Madhavan et al. [10].

To determine the cell-type composition of a hCS, we used ICC-images to manually count the number of cells based on the number of DAPI-stained nuclei and quantified the percentage of cells per field positive for PAX6 (neural progenitors; n = 3 spheroids, a total of 1370, 838 and 627 cells counted), Vesicular glutamate transporter 1 (VGLUT1; excitatory neurons; n = 3 spheroids, a total of 1794, 542 and 895 cells counted), Glutamate Decarboxylase 1 (GAD1; inhibitory neurons; n = 3 spheroids, a total of 1401, 810, and 522 cells counted), Glial Fibrillary Acidic Protein (GFAP; astrocytes; n = 4 spheroids, a total of 1377, 846, 1277 and 1451 cells counted), O1 (OPC; n = 4 spheroids, a total of 1699, 1400, 837 and 1227 cells counted), CC1 (OPC; n = 2 spheroids, a total of 1071 and 568 cells counted), Myelin-basic protein (MBP; pre-myelinating oligodendrocytes; n = 3 spheroids, a total of 767, 1282 and 772 cells counted), Transmembrane Protein 119 (TMEM119; microglia; n = 4 spheroids, a total of 1055, 1389, 761 and 814 cells counted) and Platelet endothelial cell adhesion molecule 1 (PECAM1; endothelial cells; n = 3 spheroids, a total of 1099, 1148 and 811 cells counted). In view of the variations in the degree of spheroid border- and center stainings for some of these markers, for each marker staining we averaged the percentages of cells counted in the three quantified border images and those in the three quantified center images, resulting in the total number of cells reported above.

To compare the cellular composition of our d150 hCSs with those of various human fetal developmental stages and brain regions, we used the human fetal brain single-cell RNA-seq reference data sets of Zhong et al. ([33]; GSE104276) and Fan et al. ([32]; GSE103723). Unfortunately, the commonly used single-cell RNA-seq dataset of various stages of human fetal brain development [34] did not include a sufficient number of (micro)glial and endothelial cells to allow us to reliably estimate the d150 hCS cell-type composition. We grouped the sub-cell types reported by Zhong et al. [33] into six main cell types (Additional file 12; Zhong et al. did not report data for endothelial cells and pre-myelinating oligodendrocytes) and the sub-cell types from [32] into the eight main cell types that were also quantified based on our ICC-data: neural progenitor cells (NPCs), inhibitory neurons, excitatory neurons, astrocytes, oligodendrocyte precursor cells (OPCs), pre-myelinating oligodendrocytes, microglia and endothelial cells (Additional file 13). Normalization of the expression levels per cell type was performed using the median expression values of the housekeeping genes GAPDH, YHWAZ, PPIA, EIF4A1, ATP synthase subunit b (ATP5F1), Phosphoglycerate Kinase 1 (PGK1) and Actin Beta (ACTB), which were stably expressed among multiple CNS cell types, fetal CNS brain regions and fetal gestational weeks (GWs). Next, we compared the expression data of our hCSs, normalized with the median expression values of the same seven housekeeping genes, with the normalized expression data of Fan et al. [32] and Zhong et al. [33], using:

2 $$m_{n} = \frac{{E_{n,hCS} }}{{E_{n,ref} }}$$

With ${m}_{n}$ representing the relative expression for a given marker n, ${E}_{n,hCS}$ denoting the normalized expression of that marker in one of our hCS batches and ${E}_{n,ref}$ being the normalized expression of that same marker in the Zhong et al. [33] or Fan et al. [32] reference data set. As such, ${m}_{n}$ is an estimate for the fraction that a particular cell type represents in the hCS batch. Using this formula, we calculated ${m}_{n}$ for each of the cell-type-specific markers listed in Additional file 16: Table S3. We scaled the relative expressions in order for the estimates to sum up to 100%:

3 $$F_{x} = 100\% *\frac{{M_{x} }}{{\mathop \sum \nolimits_{c = 1}^{ncelltypes} M_{c} }}$$

With ${F}_{x}$ the estimated percentage of a given cell type x and M the median of all ${m}_{n}$ (see Eq. 2) for that cell type (*ncelltypes* being the number of cell types represented in the dataset). We analysed this independently for our batch-1 and batch-12 hCSs. For each estimated percentage, we calculated the distance to our ICC-percentages by subtracting ${F}_{x}$ from the corresponding percentage established by ICC quantification in our hCSs. These distance scores were squared and summed to obtain a general distance score based on all cell types. As the Zhong et al. [33] data was available per GW (8 until 26), we performed these estimates for the distinct GW time points. As the Fan et al. [32] data was available per brain region, we performed these estimates for the frontal cortex, parietal cortex, temporal cortex, occipital cortex and inferior surface. As the Zhong et al. [33] data was available per GW (8 until 26) and the Fan et al. [32] data per brain region (frontal cortex, parietal cortex, temporal cortex, occipital cortex and inferior surface, we calculated these percentages for the distinct GW time points and for the distinct brain regions, respectively. To get further insight into which of the human brain regions studied by Fan et al. [32] is most similar to our hCSs, we determined per brain region and per cell type the percentage of cell-type-specific markers that was expressed in our spheroids; this was performed for batch-1 as well as batch-12 hCS-expressed genes with an FPKM value > 0.5. In this case, the cell-type-specific markers were selected from the Fan et al. [32] dataset based on their expression level being > 10-fold higher in one cell type than in the other cell types (Additional file 8).

### Statistical analyses

For statistical analysis, Statistical Package for the Social Sciences (SPSS) IBM version 27 was used and all statistical tests were performed with a threshold of *p* > 0.05. Independent samples T-tests were performed to compare the normalized mRNA expression differences between H9 ESCs (three different passages) and d150 hCSs-1. The significances of the expression levels at various hCS time points relative to those of H9 ESCs were tested by a one-way analysis of variance (ANOVA) with Dunnett’s T3 post hoc comparisons to account for the unequal variances between the time points. For the analysis of mRNA expression differences among various H9 ESC passages and H9 and one hCS batch, one-way ANOVA with Bonferroni post hoc comparisons was used. To statistically analyse the mean ICC signal intensities per field (Microtubule-associated protein 2 (MAP2)/GFAP/O1) following omission of dual SMAD inhibition (-SMAD) or inclusion of dual SMAD inhibition (+ SMAD), we first performed a one-way ANOVA omnibus test for the difference between the -SMAD and + SMAD groups (F = 8.728, *p* = 0.009). Subsequently, we performed a one-way ANOVA per time point for the effect between the -SMAD or the + SMAD groups (n = 1–4; one batch). The effects in the BMP4 modulation experiments (0.1 μM DM, 1 μM DM and 2.5 μM DM) were tested with a one-way ANOVA with Tukey post hoc comparisons per time point for the TMEM119 mRNA expression levels, and MAP2, GFAP, and P2RY12 protein expression levels. The mRNA expression differences between untreated and LPS-, TNFα- and IL-1β-treated hCSs were tested with independent samples T-tests.

## Results

### Generation of neuroectoderm-derived cell types in hCSs produced from hESCs

#### Induction of neuroectoderm development

**Fig. 1:**
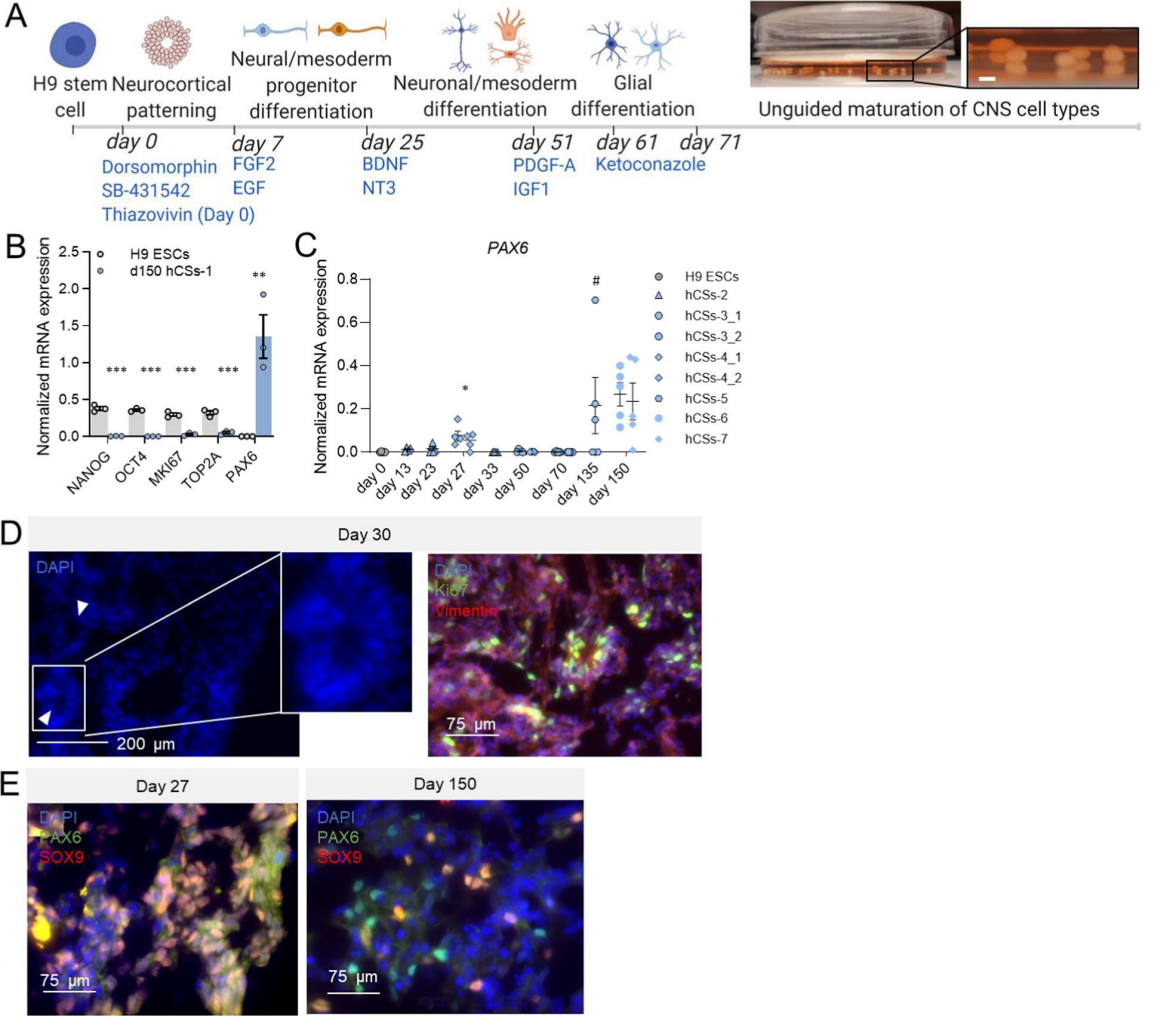
Development of neuroectoderm in H9 embryonic stem cell (ESC)-derived human cortical spheroids (hCSs) cultured in the presence of low levels of BMP4 (mild SMAD inhibition) during neurocortical patterning. **A** Schematic overview of the hCS culturing protocol, depicting crucial proteins and small molecules that induce central nervous system (CNS) cell-type differentiation. The diameters of the generated day-150 (d150) hCSs were between 0.5 and 1.5 mm; scale bar: 1 mm. **B** Levels of mRNA expression of the stem-cell-specific transcripts *NANOG* and *OCT4*, the proliferation-related transcripts *MKI67* and *TOP2A*, and the neural NPC-restricted transcript *PAX6* in H9 ECSs and d150 hCSs-1 (batch-1), as determined by quantitative PCR (qPCR) analysis. Independent samples T-test: ***p < 0.001, **p < 0.01. **C** Levels of mRNA expression of *PAX6* in H9 ESCs (d0) and hCSs at various culturing time points of several batches (hCSs2-7). Significances are relative to d0. ANOVA with Dunnett’s T3 post hoc comparisons: *p < 0.05, ^#^*p* < 0.1. Error bars represent the standard error of the mean. Each data point in (**B**, **C**) represents the level of mRNA expression in one spheroid. **D** Representative immunocytochemistry (ICC) images of neural rosettes (white arrows) containing proliferating KI67-positive cells in d30 hCSs. **E** Representative ICC-images of PAX6- and SOX9-positive cells in co-stained early (d27) and late (d150) hCSs. The thresholds of the individual color channels in (**D**, **E**) were adjusted to better represent the individual signals in the merged image

We presumed that a reduced concentration of the BMP4-signaling inhibitor DM may permit the development of mesoderm, and its subsequent differentiation to microglia and endothelial cells in neuroectoderm-guided hCSs. We therefore cultured H9 ESCs in the presence of a low concentration of DM (1 μM) during neurocortical patterning in the hCSs (d0 to d7; Fig. 1A). We first explored whether the reduction in BMP4 inhibition interferes with neuroectoderm development by performing qPCR analysis to examine the mRNA expression of a number of stem-cell, neural proliferation and NPC markers. The ESC-specific transcripts Nanog Homeobox (*NANOG;* t = 14.26, *p* < 0.001) and Octamer-binding transcription factor 4 (*OCT4;* t = 26.75, *p* < 0.001) were expressed only in H9 ESCs and not in d150 hCSs. NPCs had developed in our hCSs since the NPC marker *PAX6* was induced in d150 hCSs compared to H9 ESCs (t = 4.56, *p* = 0.005) (Fig. 1B), presumably because of the presence of extracellular matrix (ECM) components in the culture medium [35, 36], and hCS batches studied at d27 (*p* = 0.039) and at d135 (*p* = 0.089) showed a peak of *PAX6* expression (Fig. 1C). The presence of Marker of proliferation Ki-67 (MKI67)-positive proliferating cells in neural rosettes at d30 corroborated the formation of neuroectoderm in our hCSs (Fig. 1D). The occurrence of such neural rosettes during the first three months in culture rendered the structure of the developing spheroids less compact when compared to the structure of d150 hCSs. The existence of PAX6-positive neural stem cells in both still-developing d27 hCSs and more fully developed d150 hCSs (Fig. 1E) confirmed that NPC populations remained present even after neural differentiation. Not all SRY-Box Transcription Factor 9 (SOX9)-positive stem cells, which can develop into neuroectoderm, mesoderm and endoderm cells [37], displayed PAX6 expression at d27 (Fig. 1E) and we therefore conclude that stem cells that are not (yet) guided into an embryonic lineage, or ectoderm progenitors that are PAX6-negative [38], were present at this time point. Consistent with this, mRNA expression of the cell-division-related markers *MKI67* (t = 10.45, *p* < 0.001) and DNA Topoisomerase II Alpha (*TOP2A;* t = 8.52, *p* < 0.001) was decreased, but still detectable, in d150 hCSs (Fig. 1B), suggesting that at least a number of proliferating, undifferentiated cells persisted at d150. The high mRNA expression levels of *NANOG* and *OCT4*, and the low mRNA expression of *PAX6* and the mature neuronal marker Neurofilament Light Chain (*NEFL*) in H9 ESCs substantiated that our H9 passaging protocol kept the stem cells in a pluripotent state (Additional file 1: Fig. S1). These data show that reduced inhibition of BMP4 signaling still allowed the initiation of neuroectoderm development in H9-ESC-derived hCSs.

#### Induction of neuronal marker expression

**Fig. 2:**
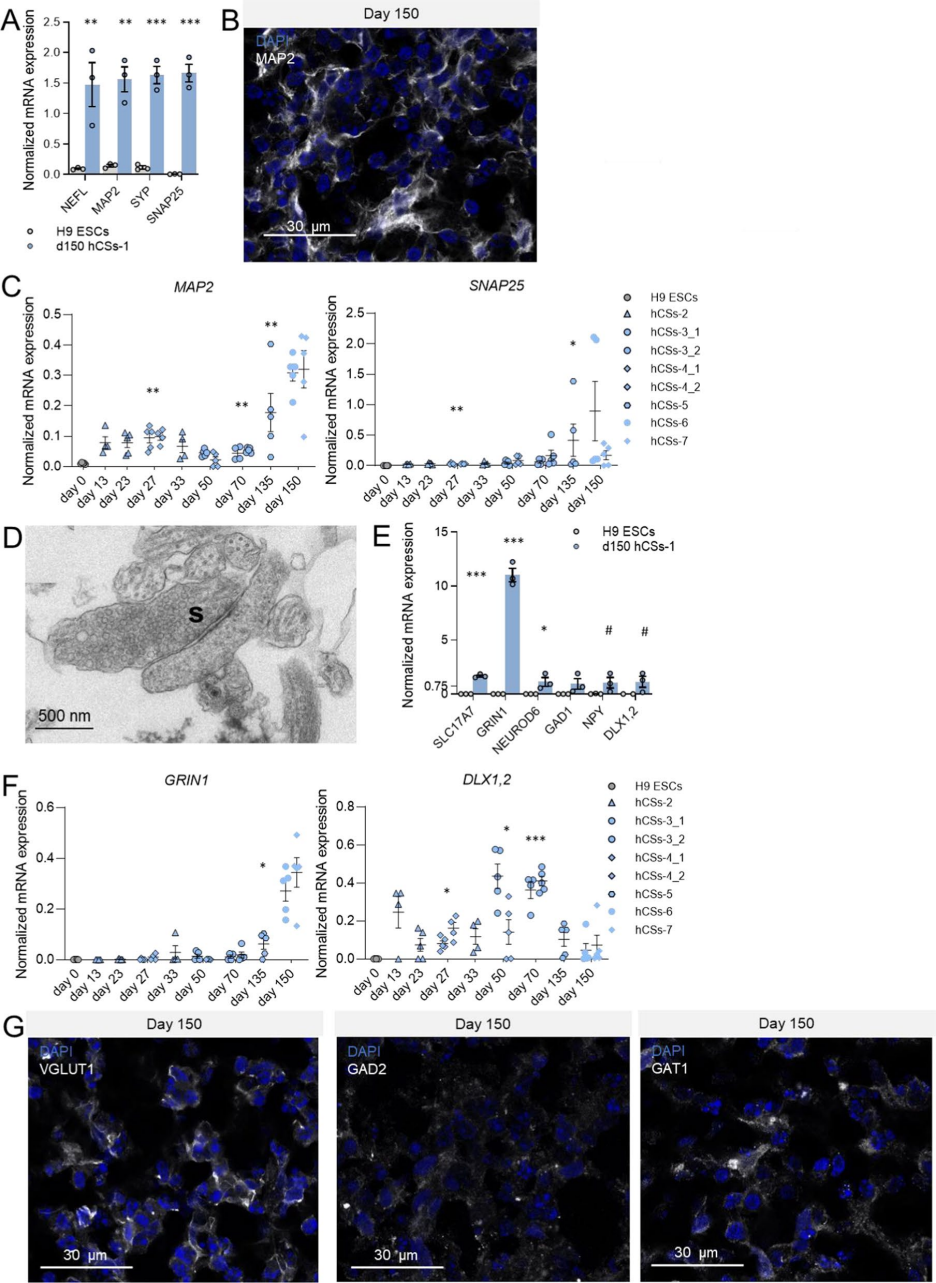
Generation of mature neuronal subtypes and synaptic structures in human cortical spheroids (hCSs). **A** Levels of mRNA expression of the mature neuronal markers *NEFL* and *MAP2*, and of the synapse-associated transcripts *SYP* and *SNAP25* in H9 embryonic stem cells (ESCs) and day-150 (d150) hCSs. **B** Representative confocal image of cells positive for the dendritic protein MAP2 in d150 hCSs (batch-1). Independent samples T-test: ***p < 0.001, **p < 0.01. **C** Levels of mRNA expression of *MAP2* and *SNAP25* in H9 ESCs (d0) and hCSs at various culturing time points. Significances are relative to d0. ANOVA with Dunnett’s T3 post hoc comparisons: **p < 0.01, *p < 0.05. **D** Representative ultrastructural transmission electron microscopy (TEM) image of a synapse (S) in d150 hCSs. **E** Levels of mRNA expression of the excitatory neuron markers *SLC17A7*, *GRIN1* and *NEUROD6*, and the inhibitory neuron-related markers *GAD1*, *NPY* and *DLX1,2* in H9 ESCs and d150 hCSs (batch-1). Independent samples T-test: ***p < 0.001, *p < 0.05, ^#^*p* < 0.1. **F** Levels of mRNA expression of *GRIN1* and *DLX1,2* in H9 ESCs (d0) and hCSs at various culturing time points. Significances are relative to d0. ANOVA with Dunnett’s T3 post hoc comparisons: ***p < 0.001, *p < 0.05. Error bars represent the standard error of the mean. Each data point in (**A**, **C**, **E**, **F**) represents the level of mRNA expression in one spheroid. **G** Representative confocal images of cells positive for the excitatory neuronal marker VGLUT1, and the inhibitory neuron markers GAD2 and GAT1 in d150 hCSs

Neurons and glial cells differentiate from neuroectodermal germ layer progenitor cells and represent the majority of cell types found in the human brain [39]. In d150 hCSs compared to H9 ESCs, we observed a 10- to 20-fold increase in the mRNA expression levels of the mature neuronal markers *NEFL* (t = 3.84, *p* = 0.009) and *MAP2* (t = 6.83, *p* = 0.001) (Fig. 2A), indicating that mature neuronal populations were present in our d150 hCSs. This was confirmed by the occurrence of MAP2-positive cells and dendritic innervations at d150 (Fig. 2B), which for the most part were evenly distributed over the spheroid. Furthermore, the mRNA expression levels of the synaptic proteins Synaptophysin (*SYP*; t = 10.52, *p* < 0.001) and Synaptosome Associated Protein 25 (*SNAP25*; t = 11.38, *p* < 0.001) were highly induced in d150 hCSs compared to H9 (Fig. 2A). Higher *MAP2* expression was found in hCS batches studied at d27 (*p* = 0.001) hCSs, and higher *SNAP25* expression was detected in the batches examined at d27 (*p* = 0.001) and at d135 (*p* = 0.048) relative to H9 ESCs (Fig. 2C). Ultrastructural studies showed the presence of synaptic vesicles already at d39 (Additional file 2: Fig. S2A) and mature synapses with dense-core vesicles at d150 (Fig. 2D; Additional file 2: Fig. S2A), a characteristic of fully matured neurons. At the ultrastructural level, we further observed that neurite outgrowth increased with culture time, and that both d39- and d150 hCS neurons displayed a remarkable number of neuronal extensions that are characterized by longitudinal or cross-sectional microtubules (Additional file 2: Fig. S2B). The expression of excitatory neuron-specific transcripts Solute Carrier Family 17 Member 7 (*SLC17A7*; t = 17.47, *p* < 0.001), Glutamate Ionotropic Receptor NMDA Type Subunit 1 (*GRIN1*; t = 17.63, *p* < 0.001), and Neuronal Differentiation 6 (*NEUROD6*; t = 2.84, *p* = 0.023) was robustly increased in d150 hCSs relative to H9 expression (Fig. 2E). The expression of the mature excitatory neuron marker *GRIN1* was increased in the hCS batch studied at d135 compared to H9 ESCs (*p* = 0.031; Fig. 2F). We observed in d150 hCSs increased expression of inhibitory neuron markers *GAD1* (t = 1.913, *p* = 0.064) and Distal-Less Homeobox 1 and 2 (*DLX1,2*; t = 1.67, *p* = 0.094) (in each case, the outlier data from one spheroid prevented statistical significance) (Fig. 2E). The immature inhibitory marker *DLX1,2* showed increased expression relative to H9 in various hCS batches studied during the neuronal differentiation phase (d27, *p* = 0.011; d50, *p* = 0.039; d70, *p* < 0.001) (Fig. 2F). The presence of VGLUT1-, GAD2- and GABA transporter 1 (GAT1)-positive cells in d150 hCSs demonstrated that mature excitatory and inhibitory neuronal populations had developed (Fig. 2G). Furthermore, RNA-seq analysis of total RNA from d150 hCSs revealed expression of markers for GABAergic (Gamma-Aminobutyric Acid Type A Receptor Subunit Alpha1 (*GABRA1),* Parvalbumin (*PVALB*)), glutamatergic (Glutamate Ionotropic Receptor AMPA Type Subunit 1 (*GRIA1*)), noradrenergic (Dopamine Beta-Hydroxylase (*DBH*)), dopaminergic (Tyrosine Hydroxylase (*TH*)*,* Forkhead Box A2 (*FOXA2),* Solute Carrier Family 6 Member 3 (*SLC6A3*)*,* LIM Homeobox Transcription Factor 1 Beta (*LMX1B*)), serotonergic (FEV Transcription Factor, ETS Family Member (*FEV*)*,* 5-Hydroxytryptamine Receptor 1A (*HTR1A*)) and cholinergic (Choline O-Acetyltransferase (*CHAT*)*,* Acetylcholinesterase (*ACHE*)) neurons (Additional file 9 and Additional file 10).

#### Induction of astrocyte, OPC and pre-myelinating oligodendrocyte marker expression

**Fig. 3:**
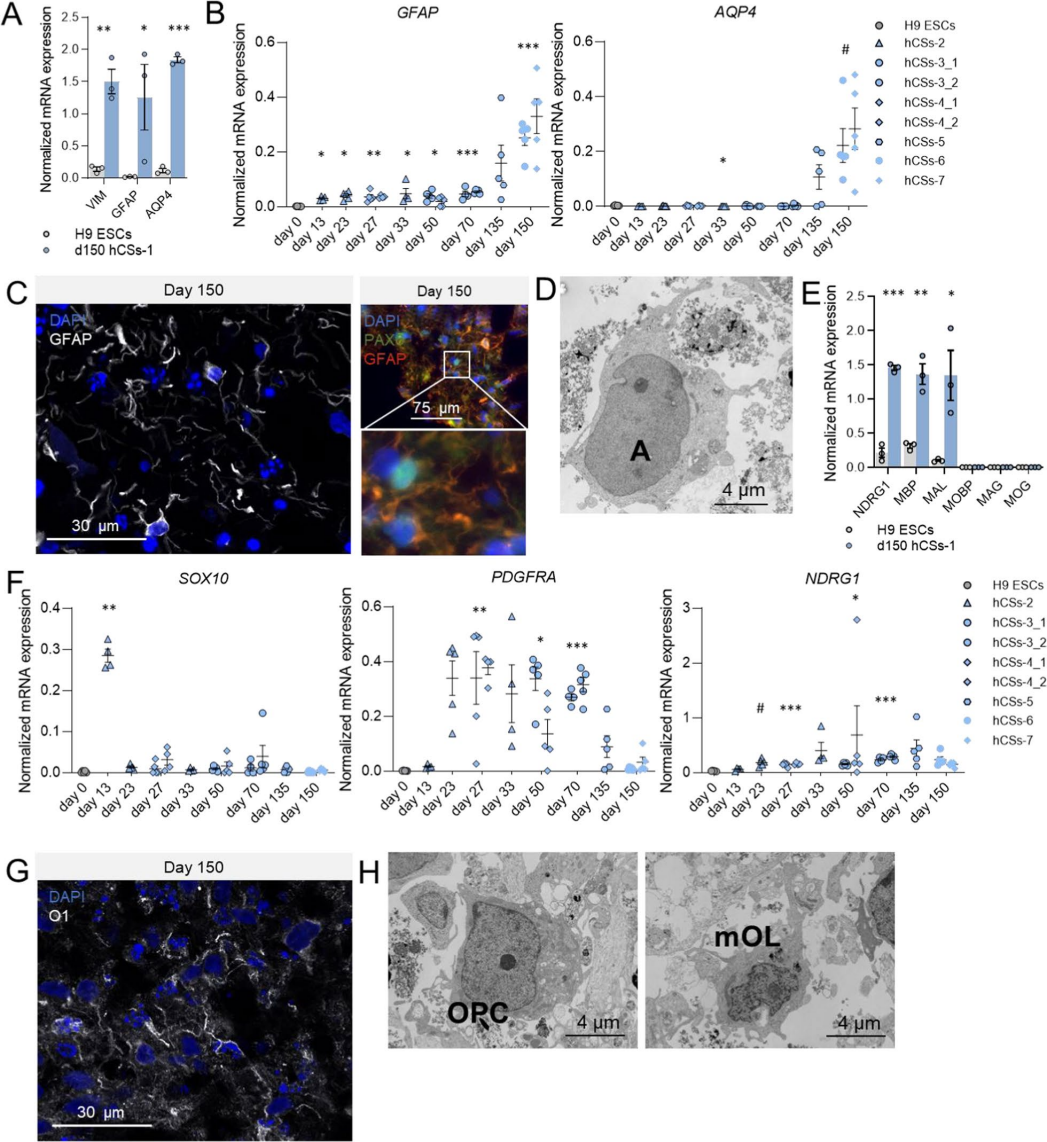
Generation of astrocytes and oligodendrocyte populations in day-150 (d150) human cortical spheroids (hCSs). **A** Levels of mRNA expression of the astrocyte-related transcripts *VIM*, *GFAP* and *AQP4* in H9 embryonic stem cells (ESCs) and d150 hCSs (batch-1). Independent samples T-test: ***p < 0.001, **p < 0.01, *p < 0.05. **B** Levels of mRNA expression of *GFAP* and *AQP4* in H9 ESCs (d0) and hCSs at various culturing time points. Significances are relative to day 0. ANOVA with Dunnett’s T3 post hoc comparisons: ***p < 0.001, **p < 0.01, *p < 0.05. **C** Left: Representative confocal image of GFAP-positive cells in d150 hCSs. Right: Double staining for GFAP and PAX6 in d150 hCSs. The thresholds of the individual color channels were adjusted to better represent the individual signals in the merged image. **D** Ultrastructural transmission electron microscopy (TEM) image of an astrocyte (**A**) in d150 hCS. **E** Levels of mRNA expression of oligodendrocyte precursor (OPC) marker *NDRG1* and mature oligodendrocyte markers *MBP*, *MAL*, *MOBP*, *MOG *and *MAG* in H9 ESCs and d150 hCSs (batch-1). Independent samples T-test: ***p < 0.001, **p < 0.01, *p < 0.05. **F** Levels of mRNA expression of* SOX10*, *PDGFRA*, and *NDRG1 *in H9 ESCs (d0) and hCSs at various culturing time points. Significances are relative to d0. ANOVA with Dunnett’s T3 post hoc comparisons: ***p < 0.001, **p < 0.01, *p < 0.05. Error bars represent the standard error of the mean. Each data point in (**A**, **B**, **E**, **F**) represents the level of mRNA expression in one spheroid. **G** Representative confocal image of cells positive for the OPC-transforming marker O1 in d150 hCSs. **H** Ultrastructural TEM images of OPC and mature oligodendrocyte (mOL) in d150 hCS

In addition to neuronal populations, the neuroectodermal germ layer gives rise to astrocytes and OPCs [40]. Following 150 days in culture and compared to H9 ESCs, we detected a 10-fold increase in mRNA expression of the astrocyte markers Vimentin (*VIM;* t = 7.05, *p* = 0.001), *GFAP* (t = 2.43, *p* = 0.036) and Aquaporin 4 (*AQP4*; t = 31.226, *p* < 0.001) (Fig. 3A). The mRNA expression levels of *GFAP* were increased in several hCS batches studied at a number of time points relative to H9 stem cells (Fig. 3B), while *AQP4* mRNA expression peaked relative to H9 ESCs in hCS batches examined at d33 (*p* = 0.029) and d150 (*p* = 0.09) (Fig. 3B). ICC stainings for GFAP showed astrocyte-like outgrowths and cells with astrocyte morphology evenly distributed at the border and the center of d150 hCSs (Fig. 3C). We realize that part of the GFAP signal may be attributed to GFAP-expressing NPC populations [41], but there was only minimal overlap between GFAP-positive cells and PAX6-positive NPCs in d150 hCSs (Fig. 3C). Ultrastructural studies on d150 hCSs confirmed the presence of cells with an irregular shape, pale cytoplasm, and pale, round and regular nuclei with condensed heterochromatin beneath the nuclear envelop (Fig. 3D; Additional file 2: Fig. S2C), all characteristics of astrocytes.

The presence of OPCs in d150 hCSs was evident from the 7-fold increase in mRNA expression of the OPC marker N-Myc Downstream Regulated 1 (*NDRG1*; t = 15.85, *p* < 0.001) compared to its expression in H9 ESCs (Fig. 3E), with its expression peaking in the hCS batches studied at d27 (*p* < 0.001), d50 (*p* = 0.03) and d70 (*p* < 0.001) (Fig. 3F). The expression of another OPC marker, Platelet Derived Growth Factor Receptor Alpha (*PDGFRA*), peaked in the hCS batch examined at d27 (*p* = 0.003), and in another hCS batch studied at d50 (*p* = 0.016) and d70 (*p* < 0.001) (Fig. 3F). The early peak of SRY-Box Transcription Factor 10 (*SOX10*) oligodendrocyte lineage cell expression in the hCS batch examined at d13 (*p* = 0.004; Fig. 3F) may indicate that early on during neuroectoderm development oligodendrocyte precursors are already formed. OPCs differentiate into pre-myelinating oligodendrocytes which in their mature state execute the myelination of axons [42]. Antibody O1, which detects a glycolipid antigen on the surface of maturing oligodendrocytes, showed in d150 hCSs immuno-positive cells (Fig. 3G), which were more localized to the border of the spheroid than to the center. Also, transcripts of the mature oligodendrocyte markers *MBP* (t = 6.77, *p* = 0.001) and Myelin And Lymphocyte Protein (*MAL*; t = 3.41, *p* = 0.013) were significantly upregulated at d150, compared to their expression in H9 ESCs (Fig. 3E). At the subcellular level, we indeed observed in d150 hCSs both OPCs (characterized by irregularities in the nuclei and many small mitochondria) and more mature oligodendrocytes (irregularly shaped nuclei and dark inclusions in the cytoplasm) (Fig. 3H; Additional file 2: Fig. S2D and S2E). We found in d150 hCSs no mRNA expression of the myelin-sheath-related markers Myelin Associated Oligodendrocyte Basic Protein (*MOBP*), Myelin Oligodendrocyte Glycoprotein (*MOG*) and Myelin Associated Glycoprotein (*MAG*) (Fig. 2E) and no signs of fully compacted myelin at the ultrastructural level. Thus, even though our d150 hCSs contained maturing oligodendrocyte cells, at this time point an obvious formation of condensed myelination around axons had not yet taken place.

### Generation of mesoderm-derived cell types in hCSs produced from hESCs

#### Induction of resting-state M0 and pro-inflammatory M1 microglia marker expression

**Fig. 4:**
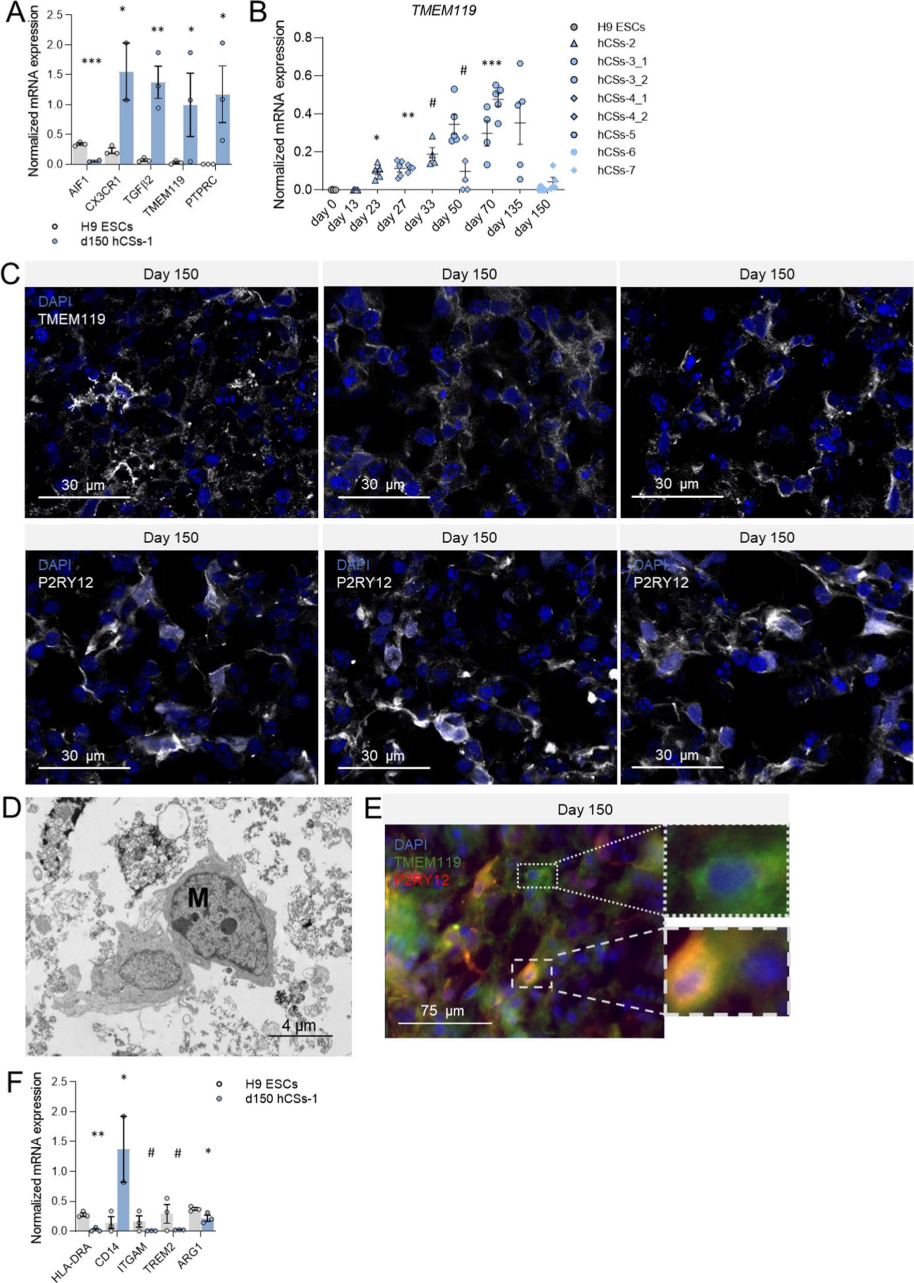
Generation of innately developing microglia subpopulations in neuron-, astrocyte- and oligodendrocyte-containing day-150 (d150) human cortical spheroids (hCSs). **A** Levels of mRNA expression of (resting-state) M0 microglia markers *AIF1, CX3CR1, TGFβ2, TMEM119* and *PTPRC *in H9 embryonic stem cells (ESCs) and d150 hCSs (batch-1). Independent samples T-test: ***p < 0.001, **p < 0.01, *p < 0.05. **B** Levels of mRNA expression of *TMEM119 *in H9 ESCs (d0) and hCSs at various culturing time points. Significances are relative to d0. ANOVA with Dunnett’s T3 post hoc comparisons: ***p < 0.001, **p < 0.01, *p < 0.05. **C** Representative confocal image of morphologies in cells positive for the pan-microglia marker TMEM119 and the M0 microglia marker P2RY12 in d150 hCSs. **D** Ultrastructural transmission electron microscopy (TEM) image of a microglia cell (M) in d150 hCS. **E** Double immunocytochemistry shows both TMEM119-positive/P2RY12-positive microglia and TMEM119-positive/P2RY12-negative cells. **F** Levels of mRNA expression of M1/M2 microglia marker *HLA-DRA*, M1 microglia marker *CD14* and M2 phagocytosis-related markers *ITGAM*, *TREM2* and *ARG1* in H9 ESCs and d150 hCSs (batch-1). Independent samples T-test: ***p < 0.001, **p < 0.01, ^#^*p* < 0.1. Error bars represent the standard error of the mean. Each data point in (**A**, **B**, **F**) represents the level of mRNA expression in one spheroid

Microglia arise from embryonic mesoderm progenitor cells. As the development of the mesodermal germ layer is more heavily guided by BMP4 than transforming growth factor-β (TGF-β) [28, 29, 43], we anticipated that a reduction in the degree of BMP4 inhibition at the start of the culturing period may allow for the simultaneous development of the neuroectodermal and mesodermal germ layers. Reduced BMP4 inhibition indeed led to increased expression of the M0 microglia transcripts C-X3-C Motif Chemokine Receptor 1 (*CX3CR1*; t = 3.61, *p* = 0.018), *TGFB2* (t = 4.796, *p* = 0.004) and *TMEM119* (t = 3.56, *p* = 0.035), and induced expression of M0 marker Protein Tyrosine Phosphatase Receptor Type C (*PTPRC*; t = 2.48, *p* = 0.034) in d150 hCSs compared to H9 ESCs (Fig. 4A). Expression of the M0 marker *AIF1*/*IBA1* (t = 10.43, *p* < 0.001) was low in d150 hCSs when compared to the also previously observed [44] surprisingly high expression in H9 ESCs (Fig. 4A). Compared to H9, the mRNA expression of *TMEM119* was already increased in the hCS batch studied at d23 (*p* = 0.05) and peaked in another batch at d70 (*p* < 0.001) (Fig. 4B), indicating that, like neurons, microglia are early-developing cells in the spheroids. Cells with surface expression of TMEM119 were detected in d150 hCSs, showing various degrees of outgrowths, often in patch-like or clustered structures (Fig. 4C).

Although the level of mRNA expression of the M0 marker *P2RY12* was low (Additional file 9 and Additional file 10), we clearly observed cell-surface P2RY12-positive cells in d150 hCSs, again with diverse degrees of outgrowths (Fig. 4C). In addition, TEM analysis revealed in d150 hCSs typical microglia-like cells with irregular contours of their cell bodies, dark amorphous inclusions in the cytoplasm, long cisternae of endoplasmic reticulum, and dark, oval-shaped nuclei containing clumped chromatin and nucleoli (Fig. 4D; Additional file 2: Fig. S2F). Together, the results indicate that populations of resting-state microglia had developed in our hCSs from an early time point onwards.

Resting-state (M0) microglia (P2RY12-positive cells) were present in 5 independently grown batches of hCSs that were analysed following 150 days of culture (Additional file 3: Fig. S3A). A low degree of variability in microglial P2RY12 protein signal intensity was found among various hCSs grown within one batch and between the 5 independent hCS batches; only batch-10 showed a significantly higher P2RY12 signal than batch-7 (*p* = 0.022) and batch-9 (*p* = 0.001) (Additional file 3: Fig. S3A). The P2RY12 protein signal intensity was independent of the starting H9 ESC cell density (Additional file 3: Fig. S3B), indicating that our protocol allows for robust development of microglia cells irrespective of the number of start cells.

A variety of microglia subtypes occur in vivo, often divided into M0, pro-inflammatory (M1) and anti-inflammatory (M2) phenotypes [45]. We found TMEM119-positive cells that either did or did not express the M0-specific P2RY12 protein (Fig. 4E), indicating that phenotypes other than M0 may be present in our hCSs. The about tenfold increased expression of the M1 microglia-associated transcript Cluster of differentiation 14 (*CD14*; t = 2.85, *p* = 0.032) in d150 hCSs relative to H9 ESCs suggested the presence of the M1 phenotype (Fig. 4F). Nevertheless, expression of Major Histocompatibility Complex, Class II, DR Alpha (*HLA-DRA*), a marker proposed for pro-inflammatory-microglia, was decreased in d150 hCSs (t = 7.14, *p* = 0.001; Fig. 4F). However, HLA-DRA expression may reflect not only an M1 but also an M2a or even “M3” phenotype [46]. Furthermore, we found low levels of the M2 microglia-related markers Integrin Subunit Alpha M (*ITGAM*)*,* Triggering Receptor Expressed On Myeloid Cells 2 (*TREM2*) and Arginase 1 (*ARG1*) in d150 hCSs (Fig. 4F), but the higher expression of these M2 markers in H9 ECSs relative to d150 hCSs (*ITGAM*: t = 1.568, *p* = 0.1; *TREM2*: t = 1.73, *p* = 0.08; *ARG1*: t = 2.783, *p* = 0.025; Fig. 4F) suggests that these proteins may exert (yet unknown) embryonic functions. Together, these data suggest that various microglial subtypes are present in the d150 hCSs.

#### Induction of endothelial lineage cell marker expression

**Fig. 5:**
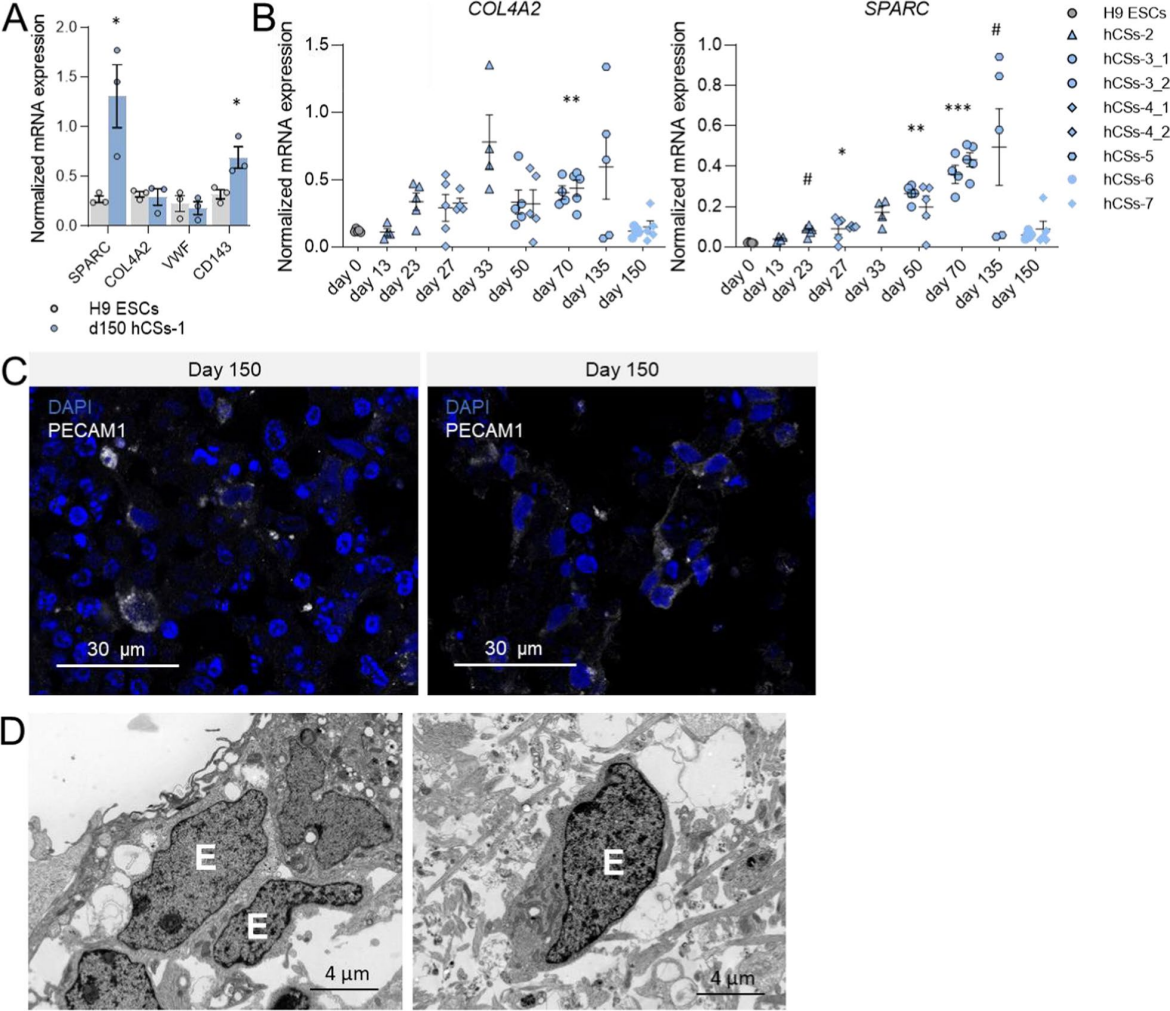
Generation of innately developing endothelial lineage cell populations in neuron-, astrocyte-, oligodendrocyte- and microglia-containing day-150 (d150) human cortical spheroids (hCSs). **A** Levels of mRNA expression of endothelial lineage transcripts *SPARC*, *COL4A2*, *VWF* and *CD143* in H9 embryonic stem cells (ESCs) and d150 hCSs (batch-1). Independent samples T-test: *p < 0.05. **B** Levels of mRNA expression of *SPARC* and *COL4A2* in H9 ESCs (day 0) and hCSs at various culturing time points. Significances are relative to d0. ANOVA with Dunnett’s T3 post hoc comparisons: ***p < 0.001, **p < 0.01, *p < 0.05, ^#^*p* < 0.1. Error bars represent the standard error of the mean. Each data point in (**A**, **B**) represents the level of mRNA expression in one spheroid. **C** Representative confocal images of PECAM1-positive cells in d150 hCSs. **D** Ultrastructural transmission electron microscopy (TEM) images of endothelial-like cells (E) in d150 hCSs

Like microglia, endothelial lineage cells differentiate from mesoderm during fetal brain development [47] and we therefore hypothesized that culturing of spheroids exposed to low BMP4 inhibition may lead to the emergence of endothelial cells. The increase in mRNA expression of the endothelial-specific transcripts Secreted Protein Acidic And Cysteine Rich (*SPARC*; t = 3.25, *p* = 0.0157) and Angiotensin-converting enzyme (*CD143*; t = 3.13, *p* = 0.018) in d150 hCSs compared to H9 ESCs indicated that endothelial cells had indeed developed in the spheroids at d150 (Fig. 5A). Compared to H9 stem cells, mRNA expression of *SPARC* was increased in the hCS batch studied at d27 (*p* = 0.025) as well as in another hCS batch examined at d50 (*p* = 0.001) and d70 (*p* < 0.001), suggesting that endothelial cells develop early during hCS culturing (Fig. 5B). Expression of the endothelial cell markers Collagen Type IV Alpha 2 Chain (*COL4A2*; t = 0.31, *p* = 0.386) and Von Willebrand Factor (*VWF*; t = 0.446, *p* = 0.339) was not increased in d150 hCS compared to H9 ESCs (Fig. 5A), although *COL4A2* expression was higher at d70 (*p* = 0.001) (Fig. 5B). Clusters of cells positive for the membrane-bound endothelial marker PECAM1 were present in d150 hCSs (Fig. 5C), even though this cell-surface marker displayed low expression at the mRNA level (Additional file 9 and Additional file 10). In line with these expression studies, TEM-analysis showed cells that strongly resembled endothelial cells, while blood vessel-like structures were not observed in d150 hCSs (Fig. 5D; Additional file 2: Fig. S2G).

Since hematopoietic stem cells are also derived from the mesodermal germ layer [48], we examined in d150 hCSs the mRNA expression levels of typical markers for T-cells (Cytotoxic And Regulatory T Cell Molecule, *CRTAM*, which is also expressed in neuronal subpopulations [49]), B-cells (Cluster of differentiation 80 (*CD80*) and Membrane Spanning 4-Domains A1 (*MS4A1*)), natural killer cells (Fc Gamma Receptor IIIa (*FCGR3A*)), leukocytes (Selectin L (*SELL*)) and dendritic cells (Cluster of differentiation 209 (*CD209*)). Except for a significantly increased level of *CRTAM* mRNA expression in d150 hCS compared to H9 ESCs (t = 2.47, *p* = 0.034), we found no expression of the other immune markers in d150 hCSs nor in H9 (Additional file 2: Fig. S2H). Because certain neuronal subpopulations are known to express CRTAM [49], it is conceivable that in our hCSs this T-cell marker is expressed by neurons rather than by immune cells. We further found a low number of mesoderm-derived muscle cells and cartilage (Additional file 2: Fig. S2I and S2J), illustrating that mesoderm-derived cell types had indeed developed in the d150 hCSs.

Together, the results indicate that our protocol allows for the development of microglia and endothelial cells that are derived from mesoderm progenitors.

#### Modulation of the degree of BMP4 inhibition alters the balance between neuroectoderm and mesoderm lineage development

When grown in the absence of dual SMAD inhibition during the first seven days (i.e., complete omission of both DM and the TGF-β inhibitor SB-431542 from the culture medium), hCSs showed a time course (d17, d33 and d73) of essentially no increase in neuronal MAP2 and astrocyte GFAP protein expression, clearly distinct from the time-dependent increased expression of these markers in hCSs grown in the presence of 1 μM DM and 10 μM SB-431542 (mild SMAD inhibition) (Additional file 4: Fig. S4A and S4B). The time course of O1-positive oligodendrocyte lineage cells development was essentially the same in the presence or absence of mild SMAD inhibition (Additional file 4: Fig. S4A and S4B). Moreover, when during the first seven days of culture we varied the degree of BMP4 inhibition at a fixed degree of TGF-β inhibition (10 μM SB-431542) the neuroectoderm-mesoderm ratio in hCSs changed. hCSs cultured in the presence of 0.1 μM DM contained no translucent neural rosette-like structures and displayed a remarkably larger size than those cultured with 1 μM DM (our standard protocol of mild SMAD inhibition) or 2.5 μM DM (Additional file 5: Fig. S5A and S5B). Neuroectoderm-derived, MAP2-positive neurons were more present in 2.5 μM DM hCSs compared to 1 μM DM hCSs at d13 (*p* = 0.001) and 0.1 μM DM at all three time points (d13: *p* = 0.002; d23: *p* = 0.042; d33: *p* = 0.041) (Additional file 5: Fig. S5C). The GFAP signal intensity was significantly lower in the 0.1 μM DM hCSs compared to that in 1 μM DM hCSs (d13: *p* = 0.002; d23: *p* = 0.001) and 2.5 μM DM hCSs (d13: *p* = 0.006; d23: *p* = 0.017) (Additional file 5: Fig. S5C). In contrast, the 0.1 μM DM hCSs showed a significantly higher level of *TMEM119* mRNA expression than 2.5 μM DM hCSs at all three time points (d13: *p* = 0.001; d23: *p* = 0.002; d33: *p* = 0.001) (Additional file 5: Fig. S5D). Thus, a higher degree of neuroectoderm was formed in 2.5 μM DM and 1 μM DM hCSs than in spheroids grown with lower or no BMP4 inhibition, while mesoderm formation dominated in hCSs cultured with 0.1 μM or in the absence of DM. These results are in line with our presumption that higher levels of BMP4 inhibition result in a propensity towards neuroectoderm development and that finetuning of the degree of BMP4 inhibition allows for the generation of both neuroectoderm- and mesoderm-derived cell types in hCSs.

### Analysis of hCS cell-type composition

**Fig. 6:**
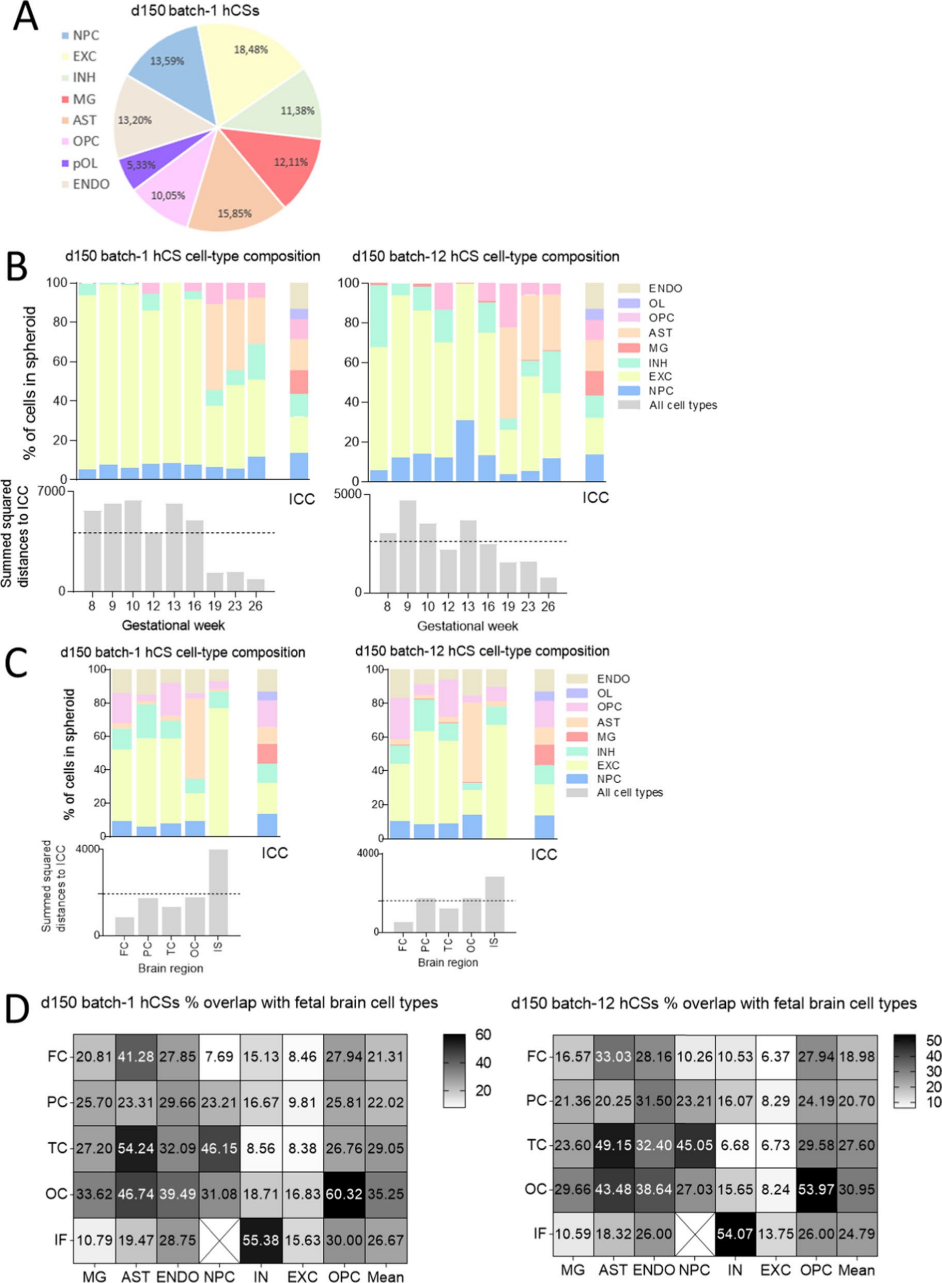
Comparative analysis of the cell-type composition of day-150 (d150) human cortical spheroids (hCSs), and the cell-type compositions of human fetal brain at various gestational weeks (GWs) and fetal brain regions. **A** Cell-type composition of d150 hCSs (batch-1) as quantified via immunocytochemistry (ICC) analysis of cell-type markers. **B** Percentages estimated per cell type in d150 hCSs (batch-1 and batch-12) based on the single-cell-type cluster-specific markers in GW8 to GW26 prefrontal cortex as deduced from the RNA-seq data of Zhong et al. [33]. **C** Percentages estimated per cell type in d150 hCS (batch-1 and batch-12) based on the single-cell-type cluster-specific markers in frontal cortex (FC), parietal cortex (PC), temporal cortex (TC), occipital cortex (OC) and the inferior surface (IS) of the midgestational (GW22/23) human fetal brain as deduced from the RNA-seq data of Fan et al. [32]. **D** Percentages of overlap between cell-type markers per brain region and transcripts expressed =  > 0.5 FPKM in (batch-1 and batch-12) d150 hCSs. The dotted lines in (**B**, **C**) represent the median of the summed squared distance to the quantifications based on ICC. NPC: neural progenitor cell; EXC: excitatory neuron; INH: inhibitory neuron; AST: astrocyte; OPC: oligodendrocyte precursor cell; pOL: pre-myelinating oligodendrocyte; MG: microglia; ENDO: endothelial cell

We first determined the cell-type composition of our d150 hCSs on the basis of ICC images acquired with cell-type-specific markers and found that the percentages of brain cell types in the spheroids were about 13.6% NPCs, 18.5% excitatory neurons, 11.4% inhibitory neurons, 15.9% astrocytes, 10% OPCs, 5.3% mature oligodendrocytes, 12.1% microglia and 13.2% endothelial cells (Fig. 6A). Next, to get insight into the degree of hCS variability regarding cell-type composition we determined the mRNA expression levels of cell-type markers by performing bulk RNA-seq analysis of pooled total spheroid RNAs isolated from two independently grown batches of d150 hCSs (Additional file 9 and Additional file 10). Brain cell-type markers were selected on the basis of their cell-specific mRNA expression levels in the single-cell RNA-seq dataset from human fetal brain [32] (Additional file 16: Table S3). The fact that the mRNA expression levels of the markers for stem cells, NPCs, excitatory and inhibitory neurons, astrocytes, mature oligodendrocytes and endothelial cells did in general not greatly vary between the two independent hCS batches studied here (2- to 5-fold difference) (Additional file 6: Fig. S6A and S6B) suggests a relatively low degree of variability among the cell-type compositions of the spheroids (Additional file 6: Fig. S6). However, for microglia and OPC markers we found more profound differences in mRNA expression levels, with two markers (*AIF1* and *COL20A1*) even showing a > 50-fold difference in expression between the two hCS batches (Additional file 6: Fig. S6). We therefore conclude that even though in the two batches all markers used for the neuroectoderm- and mesoderm-derived cell types were consistently expressed, these batches show variability in their cell-type compositions.

We next wondered to what extent the cell-type composition of the hCSs generated with our protocol compares to the cell-type composition of hCSs generated by a protocol that is similar to ours but does not lead to the development of mesoderm. Of the various studies aimed at creating hCSs, Madhavan et al. [10] used a protocol that is closest to ours and, like the other studies, did not report the presence of innately grown mesoderm cell types, as indeed confirmed by Tanaka et al. [7] through an in silico single-cell RNA-seq analysis. We therefore compared the mRNA expression levels of the human fetal brain cell-type-specific markers (Additional file 16: Table S3) in our d150 hCSs with those in the d94 hCSs from Madhavan et al. [10] (GEO accession number GSE110006, dataset GSM2976235) (Additional file 7: Fig. S7A and S7B). The mRNA expression levels of the stem-cell markers examined, except for Spalt Like Transcription Factor 4 (*SALL4*), were higher in our d150 hCSs than in the Madhavan d94 hCSs (median fold change (FC) batch-1 = 5.5; median FC batch 12 = 17.5). Our hCSs also showed higher mRNA levels of the NPC markers *MKI67* and *PAX6* (median FC batch-1 = 2.4, median FC batch-12 = 3.9), but lower mRNA expression levels of the NPC-proliferation markers *TOP2A* and High Mobility Group Box 2 (*HMGB2)* (median FC batch-1 = 0.3, median FC batch-12 = 0.5). A set of mature excitatory neuronal markers (SATB Homeobox 2 (*SATB2*)*, GRIN1, GRIN2B*) was upregulated in our hCSs compared to the Madhavan hCSs (median FC batch-1 = 4.7, median FC batch-12 = 10.2), while the expression of immature excitatory neuronal markers (*NEUROD2, NEUROD6,* Eukaryotic Translation Initiation Factor 1B (*EIF1B*) and *SLC17A7*) was lower in our hCSs (median FC batch-1 = 0.3, median FC batch-12 = 0.3). Expression of the mature inhibitory neuronal markers *GAD1* and Phosphodiesterase 4D Interacting Protein (*PDE4DIP*) (median FC batch-1 = 1.3, median FC batch-12 = 2.1) was comparable in our d150 and Madhavan d94 hCSs, except for the higher expression of the inhibitory neuronal marker Erb-B2 Receptor Tyrosine Kinase 4 (*ERBB4*) (FC batch-1 = 11.7, FC batch-12 = 24.1), and a lower expression of the immature inhibitory neuronal markers *DLX1* and *DLX2* in our hCSs (median FC batch-1 = 0.07, median FC batch-12 = 0.5). The mRNA expression of astrocyte subtype markers (*GFAP, AQP4,* Solute Carrier Organic Anion Transporter Family Member 1C1 (*SLCO1C1*)*,* ATPase Na + /K + Transporting Subunit Alpha 2 (*ATP1A2*)*,* Aldehyde Dehydrogenase 1 Family Member L1 (*ALDH1L1*)*,* Receptor Activity Modifying Protein 3 (*RAMP3*)) was consistently upregulated in our d150 hCSs (median FC batch-1 = 10.4, median FC batch-12 = 16.1). Also, the mRNA expression levels of the OPC markers *PDGFRA,* S100 Calcium Binding Protein B (*S100B*)*,* Oligodendrocyte Transcription Factor 1 (*OLIG1*) and Peripheral Myelin Protein 2 (*PMP2*) were higher in our d150 hCSs (median FC batch-1 = 5.9, median FC batch-12 = 19.4), as were the levels of the more mature oligodendrocyte markers *MBP,* UDP Glycosyltransferase 8 (*UGT8*) and 2′,3′-Cyclic Nucleotide 3′ Phosphodiesterase (*CNP*) (median FC batch-1 = 3.0, median FC batch-12 = 1.3). Crucially, the microglia-associated transcripts *P2RY12*, *PTPRC*, *ITGAM* and Complement C1q B Chain (*C1QB*) were expressed in our d150 hCSs (Additional file 9 and Additional file 10), albeit at low levels, but not in the d94 Madhavan hCSs. Moreover, the mRNA expression level of the highly specific microglia marker *TMEM119* was much higher in our hCSs than in the Madhavan hCSs (FC batch-1 = 46.3, FC batch-12 = 270.3). Furthermore, the endothelial cell lineage markers *SPARC,* Insulin Like Growth Factor Binding Protein 7 (*IGFBP7*)*, COL4A2* and Hes Family BHLH Transcription Factor 1 (*HES1*) were more expressed in our d150 hCSs (median FC batch-1 = 1.9, FC batch-12 = 3.4). These results suggest that the cell-type composition of our hCSs deviates from that of the Madhavan hCSs, with the most notable difference being the presence of microglial and endothelial (mesoderm-derived) populations following the application of mild BMP4 inhibition in our protocol.

#### Comparative analysis of cell-type compositions of our d150 hCSs and human fetal brain gestational time points and regions

Next, we set out to analyse to what extent the cell-type composition of our d150 hCSs resembles those of various developmental stages and regions of the human fetal brain. On the basis of the single-cell RNA-seq data obtained from GW8 to 26 human fetal brains [33], we found the highest overlap between the cell-type compositions of our d150 hCSs, and those of human fetal brain GW19, GW23 and GW26, as shown by their low distance scores to our quantified cell-type composition based on ICC (Fig. 6B). In a similar fashion and based on the RNA-seq data of frontal, temporal, parietal, occipital and inferior cortex from the human fetal brain at GW22 and GW23 [32], we found that our a priori quantified cell-type compositions of the d150 hCSs (based on protein expression) were most similar to the frontal cortex of the human fetal brain and least similar to the human fetal brain inferior surface (Fig. 6C). Since in general the level of protein expression observed does not necessarily reflect RNA expression levels [50], we acknowledge that a comparison of cell-type compositions based on protein and RNA marker expression levels only provides a first impression, despite the fact that we used multiple cell-type-specific markers. Furthermore, on the basis of the single-cell data from Fan et al. [32] we determined per brain region a larger set of brain cell-type-specific markers (Additional file 8) than listed in Additional file 16: Table S3 and confirmed that our hCSs express a relatively large number of microglia and endothelial cell markers (ranging from 10 to 40% depending on the brain region) (Fig. 6D). Together, these results indicate that the cell-type composition of our human d150 spheroids is most in line with those of dorsal human fetal brain regions of GW19-26 (midgestation) and confirm that markers for mesoderm-derived cell types are expressed in these hCSs.

### Pro-inflammatory stimulation induces a neuroinflammatory response in d150 hCSs

**Fig. 7:**
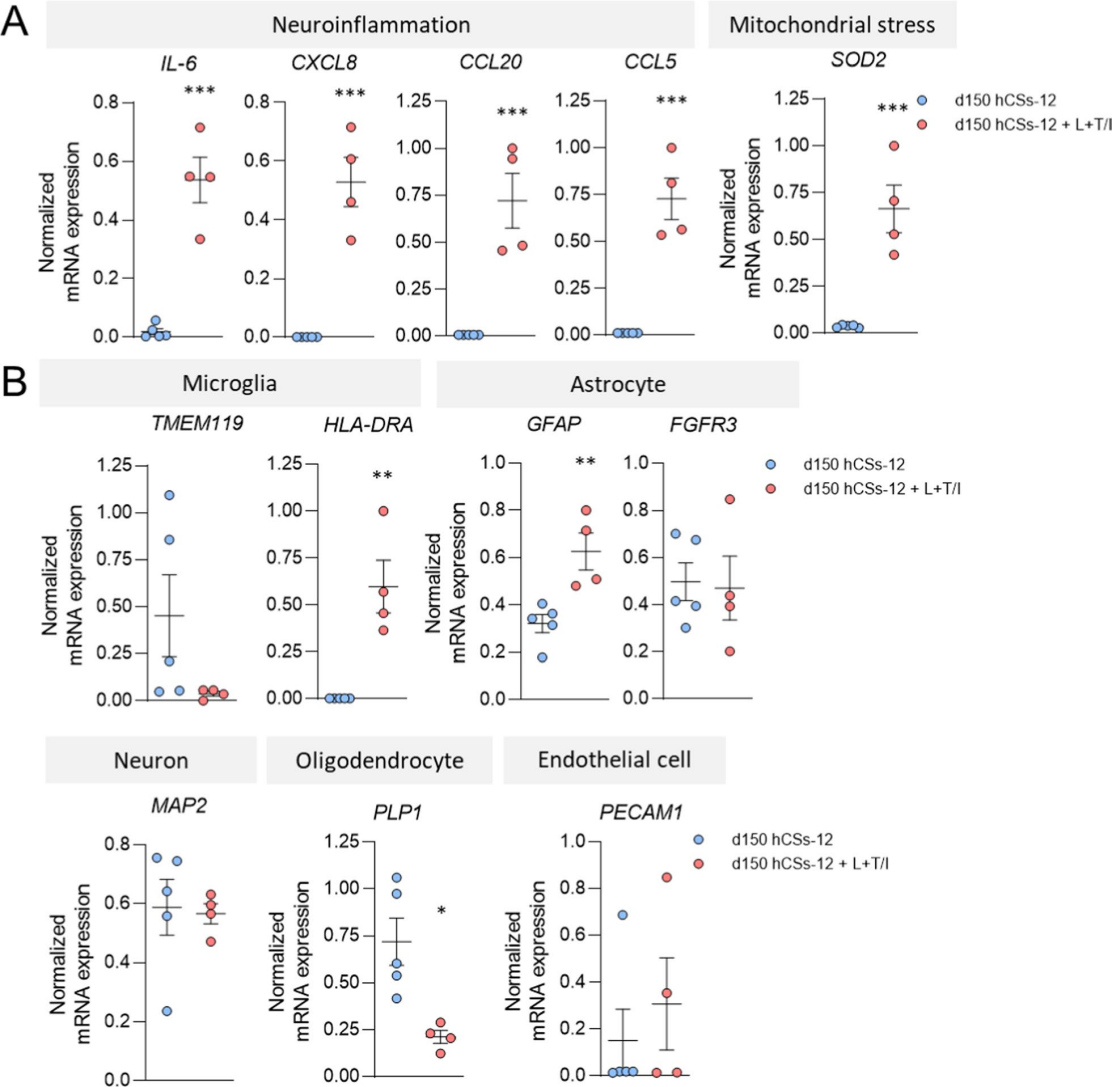
Stimulation of day-150 (d150) human cortical spheroids (hCSs) with a cocktail of pro-inflammatory factors induces a neuroinflammatory response. **A** Levels of mRNA expression of the neuroinflammatory cytokines *IL-6*, *CXCL8*, *CCL20* and* CCL5*, and the mitochondrial stress marker *SOD2* in unstimulated hCSs (batch-12) and hCSs stimulated with a cocktail of LPS (100 ng/ml, 24 h) followed by TNFα and IL1β (5 ng/ml each, 24 h) (L + T/I). Independent samples T-test: ***p < 0.001. **B** Levels of mRNA expression of cell-type-specific markers in unstimulated and stimulated hCSs (batch-12). Independent samples T-test: **p < 0.01, *p < 0.05. Error bars represent the standard error of the mean. Each data point in (**A**, **B**) represents the level of mRNA expression in one spheroid

To ascertain whether the hCSs generated with our protocol are useful to gain biological insight into human brain processes, we decided to expose d150 hCSs to a pro-inflammatory cocktail (LPS, followed by the pro-inflammatory cytokines TNFα and IL-1β). Interestingly, qPCR analysis of RNA extracted from exposed and control hCSs revealed that the mRNA expression levels of the pro-inflammatory cytokines Interleukin-6 (*IL-6;* t = 7.45, *p* < 0.001), C-X-C Motif Chemokine Ligand 8 (*CXCL8*; t = 7.15, *p* < 0.001), C–C Motif Chemokine Ligand 20 (*CCL20*; t = 6.51, *p* < 0.001) and C–C Motif Chemokine Ligand 5 (*CCL5*; t = 6.96, *p* < 0.001) as well as of the mitochondrial stress marker Superoxide Dismutase 2 (*SOD2*; t = 7.1, *p* < 0.001) were clearly induced (Fig. 7A), indicating that a neuroinflammatory response was elicited in the stimulated hCSs. Neuroinflammation is a characteristic pathological feature observed in the brains of patients with a neurological disease such as Alzheimer’s disease and multiple sclerosis [51, 52]. The mRNA levels of the microglia marker *HLA-DRA* (t = 4.39, *p* = 0.003) and the astrocyte marker *GFAP* (t = 3.77, *p* = 0.007) were also increased, while mRNA expression of the oligodendrocyte marker Proteolipid Protein 1 (*PLP1;* t = 3.4, *p* = 0.011) was decreased, and of the neuronal marker *MAP2* and the endothelial marker *PECAM1* was not affected in the pro-inflammatory-stimulated hCSs (Fig. 7B).

## Discussion

In this study, we show that culturing hESCs at a relatively low degree of BMP4 inhibition (mild SMAD inhibition) results in hCSs containing not only neuroectoderm-derived stem-cell and NPC populations, inhibitory and excitatory neurons, astrocytes and oligodendrocyte (precursor) cells, but also mesoderm-derived microglia and endothelial lineage cells. To our knowledge, our protocol is the first to produce hESC-derived hCSs with this level of cellular diversity and maturity, grown in a guided and innately developing fashion. Complete omission of dual SMAD inhibition and 10 times less BMP4 inhibition than used in our protocol during the first seven days of culture reduced the proportion of neuroectoderm-derived cell types, while 2.5 times more BMP4 inhibition than used in our protocol increased this proportion. Therefore, the degree of BMP4 inhibition determines the ratio between the extent of the development of neuroectodermal relative to mesodermal germ layers in hCSs. Since our protocol did not include the use of microglia- or endothelial lineage-cell-differentiation factors, we argue that the neuroectodermal environment created during the initial developmental stages may have encouraged the differentiation of mesodermal progenitors into microglia and endothelial cells. Indeed, secreted factors derived from neurons and glial cells have been shown to stimulate microglia- and endothelial-cell differentiation in vitro and in vivo [53].

The expression of stem-cell and NPC markers in our 5-months-old hCSs indicates that undifferentiated cell populations remained present throughout the culturing period. Since embedding of spheroids in ECM components such as laminin and collagen IV increases NPC survival [35, 36], the ECM-containing Geltrex matrix present in our hCS culture medium may have facilitated the survival of the NPC populations. Our d150 hCSs showed more stem-cell survival, and less proliferative TOP2A-positive and more early PAX6-positive NPC populations than the d94 hCSs of Madhavan et al. [10], who used a protocol most similar to ours. The human brain is also characterized by the presence of NPC subpopulations with different ratios of TOP2A and PAX6 expression [39, 54]. Interestingly, TOP2A expression often reflects a state of high NPC proliferation [54, 55], while PAX6 is considered an early-neuroectodermal fate marker [39, 56]. Furthermore, the compositions of the NPC subpopulations found in a number of single-cell RNA-seq studies on the human fetal brain appear to be variable among the various reports [32–34, 39, 57]. Yet, one has to realize that the vast heterogeneity in NPC subtypes [39], and their close relationship with intermediate progenitor cells and certain mature neuronal populations [33] complicates the designation of specific NPC fetal brain markers and thus identification of distinct NPC subpopulations.

Our d150 hCSs contained mature populations of both excitatory and inhibitory neurons. During human fetal brain development, excitatory neuron populations arise from the dorsal pallium, while γ-Aminobutyric acid (GABA)ergic inhibitory neurons are believed to originate from a more ventral pallial region, the medial ganglionic eminence [58]. Even though our growth protocol mainly induced dorsal cortical structures, we did detect GABAergic inhibitory neurons in the d150 hCSs. Interestingly, human cortical inhibitory neurons may have a dorsal origin at later developmental stages [59]. In line with a dorsal origin of excitatory neurons and the fact that in human fetal brain the development of excitatory neurons peaks at GW16 while inhibitory neuron development peaks at GW26 [33], our hCS protocol generates a somewhat higher number of excitatory than inhibitory neurons. Since our hCSs resemble frontal rather than inferior human fetal brain regions, it is interesting to note that a higher number of excitatory relative to inhibitory neurons also occurs in more frontal regions than in inferior regions of the human fetal brain [32]. The expression of synapse-specific transcripts in the d150 hCSs together with our ultrastructural data indicate that the mature neuronal populations had also initiated synaptogenic programs, consistent with the start of neuronal maturation and neocortical synaptogenesis around week 18 of human fetal brain development [33, 58]. The high degree of maturity of the neuronal subpopulations in our d150 hCSs is underscored by the higher level of expression of mature than immature neuronal markers and implies a higher neuronal maturity than that in the younger d94 Madhavan hCSs. The functionality of the neuronal populations generated with our protocol remains to be established.

In addition to the neuroectoderm-derived NPC and neuronal populations, the major pan-astrocyte transcripts GFAP, ALDH1L1 and AQP4 [60] were expressed in our d150 hCSs. Based on the mRNA expression profiles of multiple astrocyte markers, we conclude that our spheroids contain a higher degree of diversity of astrocytes than the Madhavan d94 spheroids, again consistent with the more than 50-day difference in culture-time period. Human fetal brain astrocytes indeed diversify after their first appearance around GW13-15 and continuously adapt to their environment [58, 61]. The emergence of GFAP-expressing astrocytes in the spheroids already early on coincides with the development of immature neurons and as such deviates from glial differentiation in the human fetal brain that typically occurs only from GW13 onwards and thus later than neurogenesis [58].

Application of most previous hCS protocols has not led to the expression of myelin-related transcripts such as MOBP, MAG and MOG, or the generation of fully myelinating oligodendrocytes in the spheroids [7]. The absence of compact myelin in our d150 hCSs is consistent with the findings of Madhavan et al. [10] at this culture time point. Myelination did occur in studies that used a brain spheroid culture time of 200 days [10] or cultured spinal cord spheroids in which myelination occurs much faster than in the brain [62], while [9] reported compacted myelin in hCSs at a surprisingly early culture time point of 8 weeks. We observed OPC-related and mature-oligodendrocyte marker expression in our d150 spheroids, suggesting that initial myelination programs had started. Therefore, most observations are in concordance with the findings that in the developing human brain mature oligodendrocytes are formed at a relatively late stage [58], myelinogenesis starts postnatally and the process of myelination even continues well into the postnatal period [63]. In any case, it appears that the induction of mesoderm-derived cell types through mild BMP4 inhibition does not interfere with oligodendroglial differentiation.

Early during hCS development, and concomitant with the appearance of neuronal and astrocyte populations, we observed innately grown resting-state microglial cells. Quadrato et al. [8] have reported innately grown neuroectoderm- and mesoderm-progenitor cell types in non-guided iPSC-derived hCSs, but these spheroids did not harbor mature microglia nor endothelial cells, as revealed by Tanaka et al. [7]. Ormel et al. [20] also used a non-guided protocol and, in addition to neurons and astrocytes, found innately grown mature microglia, but did not examine the presence of oligodendrocyte lineage cells and endothelial cells in their d52 hCSs. Like Ormel et al. [20] and consistent with the similar developmental time lines of neuronal cells, astrocytes and microglia during human fetal brain maturation [33], we observed a concurrent appearance of these brain cell types. Furthermore, in line with the appearance of innate microglia in spheroids grown with the non-guided protocol of Ormel et al. [20], we found increased microglial marker expression during spheroid development, the occurrence of microglia in patch-like structures and the presence of microglia with various degrees of ramification. The relatively low number of microglial cells in our d150 hCSs as well as in hCSs generated by Ormel et al. [20] is in agreement with the low percentage of microglial cells in human fetal cortical brain regions [32, 34, 64]. Since microglial populations seem to emerge in our hCSs already from around d33, the clearly lower expression of microglia-specific transcripts (such as TMEM119) in the d94 spheroids of Madhavan et al. [10] is likely due to their use of stronger BMP4 inhibition, which implies less mesoderm development.

The expression of inflammatory M1 microglia markers in our d150 spheroids complies with reports of cellular stress in hCSs due to high-energy demands, characterized by endoplasmic reticulum and ribosomal stress, phagocytosis and apoptosis [65]. The presence of necrotic cells in the center of our hCSs (based on the presence of fragmented nuclei) and in human brain spheroids from others [66] indicates that during culturing nutrient supply becomes suboptimal in spheroids with increasing size, presumably because of the absence of vascularization. Such a lack of vascularization has been one of the drawbacks of current hCS models, especially in light of the increasing role of the BBB in the pathophysiology of a variety of neurological diseases [67]. We found that mild BMP4 inhibition allows expression of multiple endothelial-cell-specific transcripts and (pro)angiogenesis-related factors as well as endothelial-specific surface expression of PECAM1 in d150 hCSs. As we did not observe blood vessel-like structures, we infer that although our PECAM1-positive endothelial cell populations appeared to be mature, vascular lumen formation had not yet occurred. The presence of mesoderm-derived proteoglycan-expressing cells destined for endothelial cell differentiation has been reported in iPSC-derived brain spheroids [8], but these cells have not been shown to innately grow in parallel with microglia and the neuroectoderm-derived cell types we describe here.

To further characterize our spheroids, we set out to compare their cell-type composition with those of various developmental stages and regions of the human fetal brain. Interestingly, the cell-type composition of our 5-months (21 weeks) old spheroids resembled the cell-type compositions of human fetal brains at GW19-26 as well as being more reminiscent of GW22/23 dorsal frontal cortical than of inferior regions. Thus, reduced SMAD inhibition appears to still result in the development of frontal cortical hCSs, as is the case with higher levels of SMAD inhibition used in other protocols [68]. Future single-cell RNA-seq analysis will allow the identification of the molecular signatures of subpopulations of each of the various brain cell types that comprise our hCSs. Furthermore, such analysis may disclose the presence of cell types other than neuronal progenitors, neurons, astrocytes, oligodendrocytes, microglia and endothelial cells, such as cartilage and muscle cells, which will affect the comparison of the cell-type composition of our spheroids with that of the fetal brain.

We observed that in the d150 hCS batches used for RNA-seq (hCS-1 and hCS-12) as well as in the d150 hCS batches used for the mRNA expression and protein expression studies (hCS-1, hCS-6 and hCS-7) neurons, astrocytes, oligodendrocyte lineage cells, microglia and endothelial cells were present, implicating that in our hCSs neuroectoderm- as well as mesoderm-derived cell types reproducibly occur in independent batches. Yet, we found variability among batches and within batches regarding the cell-type compositions of the hCSs that is likely due to the stochastic nature of in vitro self-organization and cell-fate decisions when using guided or self-organizing protocols [69]. Moreover, the number of neuroectoderm- and mesoderm-derived cell types was not always similar in the center and border of the spheroid, and also not in the z-plane, which complicated the ICC-quantifications of the cell-type compositions. Still, the innate development of both neuroectoderm-derived and mesoderm-derived brain cell types in our hCSs may better reflect the in vivo situation than human neuroectoderm-derived spheroids in which human- or rodent-derived microglia and endothelial cells have been incorporated.

In line with the neuroinflammatory response detected by Ormel et al. [20] in LPS-stimulated spheroids, our proof-of-concept study demonstrated the sensitivity of the generated hCSs towards pro-inflammatory stimulation, indicating their biological relevance. The exact brain cell type(s) driving the neuroinflammatory response in the hCSs, presumably involving the microglia and/or the astrocytes, remains to be established. Since ESCs and iPSCs appear to be molecularly and functionally equivalent [70, 71], it is conceivable that the use of our protocol will also lead to the production of innately developing neuroectoderm- and mesoderm-derived cell types in spheroids generated from other ESC lines or (patient) iPSCs. As such, our protocol will likely be instrumental for generating CRISPR-Cas9-manipulated hCSs and (patient) iPSC-derived spheroids that are valuable for recapitulating pathological features of neurological diseases. Our protocol is of particular interest in this regard as it includes the generation of microglia, astrocytes, neurons and endothelial cells, and many neurological diseases are associated with neuroinflammation and a dysfunctional neuron-immune crosstalk [72–74].

## Conclusions

We here present a culture protocol that allows the production of 3D ESC-derived hCSs with a high degree of diversity of innately developing brain cell types, providing a stepping stone for the generation of even more complex human brain spheroid models. Furthermore, these spheroids may serve as an attractive 3D model for human neurodevelopmental (disorder) studies, neurotoxicological and drug screenings, and exploring the molecular mechanisms underlying neurodegenerative diseases.

## Supplementary Information

**Additional file 1: Fig. S1**. The protocol used to culture H9 human embryonic stem cells (ESCs) does not impair their pluripotency. Levels of mRNA expression of stem-cell-specific markers *NANOG* and *OCT4*, neural stem cell marker *PAX6* and mature neuronal marker *NEFL* in H9 ESCs (six consecutive passage-numbers, P9-P14) and in day-150 (d150) batch-1 human cortical spheroids (hCSs-1). Error bars represent the standard error of the mean; ANOVA with Bonferroni post hoc comparisons: *** p < 0.001, ** p < 0.01 and * p < 0.05.

**Additional file 2: Fig. S2**. Ultrastructural transmission electron microscopy (TEM) analysis of early-stage (day-39, d39) and late-stage (d150) human cortical spheroids (hCSs) reveals multiple neuroectoderm-derived and mesoderm-derived cell types. **(A)** Left: Synapse formation in d39 hCS (yellow arrow head: synaptic vesicles). Right: Mature synapses (S) with vesicles (V) in d150 hCS (red arrow heads: dense core vesicles). **(B)** Overview images of d39 and d150 hCSs showing differences in cell density, length of outgrowths and number of cell contacts. Representative images of **(C)** astrocytes, **(D)** oligodendrocyte precursor cells (encircled in right image), **(E)** mature oligodendrocytes, **(F)** microglia cells and **(G)** endothelial cells in d150 hCSs. **(H)** Levels of mRNA expression of the T-cell/neuronal marker *CRTAM,* the B-cell markers *CD80* and *MS4A1*, the natural killer cell marker *FCGR3A,* the leukocyte marker *SELL* and the dendritic cell marker *CD209* in H9 human embryonic stem cells (ESCs) and d150 hCSs, as determined by quantitative PCR (qPCR) analysis. Independent samples T-test: * p < 0.05. Error bars represent the standard error of the mean. Each data point represents the level of mRNA expression in one spheroid. **(I)** Muscle tissue (‘Mus’) observed occasionally in d150 hCS. **(J)** Cartilage tissue (‘Cart’) observed occasionally in d150 hCS.

**Additional file 3: Fig. S3**. Low variability in the number of P2RY12-positive microglial cells among independent human cortical spheroid (hCS) batches and in hCSs grown with various start-cell densities. **(A)** Left: Representative immunocytochemistry (ICC) images of whole-spheroid signal scans of the microglia marker P2RY12 in five independently grown batches of day-150 (d150) hCSs. Microglia often developed in patch-like structures within the hCSs. Right: Quantifications of the mean P2RY12 signal intensities per field in five independently grown d150 hCS batches. ANOVA with Tukey post hoc comparisons: ** p < 0.01, * p < 0.05. **(B)** Left: Representative ICC images of whole-spheroid P2RY12 signal scans of d150 hCSs grown with various start H9 human embryonic stem cell (ESC) densities. Right: Quantifications of the mean P2RY12 signal intensities per field in d150 hCSs grown with 1.0*10^4^, 2.0*10^4^ or 4.0*10^4^ start H9 ECSs. ANOVA with Tukey post hoc comparisons: ns, not significant. Each data point in **(A)** and **(B)** represents the level of mRNA expression in one spheroid. Error bars represent the standard error of the mean.

**Additional file 4: Fig. S4**. Complete omission of SMAD inhibition (absence of dorsomorphin and SB43152 in the culture medium) results in mitigated growth of neuroectoderm-derived neurons and astrocytes. **(A)** Representative images of whole-spheroid signal scans of the neuronal marker MAP2, the astrocyte marker GFAP and the oligodendrocyte marker O1 in day-17 (d17), d33 and d73 human cortical spheroids (hCSs) (batch-11) grown with dorsomorphin and SB-431542 (+ SMAD inhibition) or without dorsomorphin and SB-431542 (- SMAD inhibition) in the culture medium. **(B)** Quantifications of the mean MAP2-, GFAP- and O1-signal intensities per field in d17, d33 and d73 hCSs (batch-11) grown with or without SMAD inhibition. One-way ANOVA per time point for -SMAD *vs* +SMAD: * p < 0.05. Each data point represents the level of mRNA expression in one spheroid. Error bars represent the standard error of the mean.

**Additional file 5: Fig. S5**. Modulation of BMP4 concentration tweaks the ratio between the extent of neuroectoderm- and mesoderm development. **(A)** Representative images of the morphologies and sizes of human cortical spheroids (hCSs) (batch-2), including neuroectoderm-layer formation/neural rosettes (blue arrow heads) at days 1, 12, 23 and 70 following culturing in the presence of 10 μM SB43152 and 0.1, 1, 2.5 or 10 μM dorsomorphin (DM). hCSs cultured in the presence of a high degree of SMAD inhibition (10 μM DM) and with a starting density of 1.25*10^4^ H9 ESCs did not aggregate into spheres. **(B)** Quantifications of hCS (batch-2) circumferences during days 0 until 70 following culturing with 10 μM SB43152 and 0.1, 1 or 2.5 μM DM. **(C)** Representative immunocytochemistry whole-spheroid (batch-2) images and mean field-intensity quantifications of MAP2 (neuron) and GFAP (astrocyte) protein expression in day-13 (d13), d23 and d33 hCSs cultured in the presence of 0.1, 1 or 2.5 μM DM. ANOVA with Tukey post hoc comparisons: ** p < 0.01, * p < 0.05. **(D)** Levels of mRNA expression of the microglial marker *TMEM119* in d13, d23 and d33 hCSs (batch-2) following culturing with 10 μM SB43152 and 0.1, 1 or 2.5 μM DM, as determined by qPCR analysis. ANOVA with Tukey post hoc comparisons: ** p < 0.01, * p < 0.05. Each data point in **(C)** and **(D)** represents one spheroid. Error bars represent the standard error of the mean.

**Additional file 6: Fig. S6**. Robust presence of both neuroectoderm-derived and mesoderm-derived cell types in two independently grown day-150 (d150) human cortical spheroid (hCS) batches, with slightly different ratios in cell-type composition. **(A)** Log_10_-converted fold change (FC) of median GAPDH-normalized mRNA expression of markers for various brain cell types in batch-1 relative to batch-12 d150 hCSs. Each bar represents the median of the expression of all cell-type-specific markers for that brain cell type. **(B)** Log_10_-converted FC of median GAPDH-normalized mRNA expression of cell-type-specific markers for various brain cell types in batch-1 relative to batch-12 d150 hCSs. See Additional file 16 for markers used. SC: stem cell; NPC: neural progenitor cell; EXC: excitatory neuron; INH: inhibitory neuron; ASTR: astrocyte; OPC: oligodendrocyte precursor cell; pOL: pre-myelinating oligodendrocyte; MG: microglia; ENDO: endothelial cell.

**Additional file 7: Fig. S7**. Relative distributions of distinct cell types based on transcriptomic comparisons between day-150 (d150) human cortical spheroids (hCSs) generated with our protocol and d94 hCSs generated with the T3-induction protocol of Madhavan et al. (2018). This figure is related to figure 6. **(A)** Log_10_-converted fold change (FC) of GAPDH-normalized mRNA expression of cell-type-specific markers in d150 hCSs (batch-1) (present study) relative to that in d94 hCSs (Madhavan et al., 2018). **(B)** Log_10-_converted FC of GAPDH-normalized mRNA expression of cell-type-specific markers in d150 hCSs (batch-12) (present study) relative to that in d94 hCSs (Madhavan et al., 2018).

**Additional file 8**. Establishing cell-type markers based on the single-cell RNA-seq dataset of Fan et al. [32].

**Additional file 9**. Levels of mRNA expression (FPKM values) as determined by RNA-seq analysis of total RNA from day-150 human cortical spheroids of batch-1.

**Additional file 10**. Levels of mRNA expression (FPKM values) as determined by RNA-seq analysis of total RNA from day-150 human cortical spheroids of batch-12.

**Additional file 11**. Specificity of cell-type markers that were identified on the basis of literature search.

**Additional file 12**. Grouping strategy of sub-cell types reported by Zhong et al. [33].

**Additional file 13**. Grouping strategy of sub-cell types reported by Fan et al. [32].

**Additional file 14: Table S1**. Nucleotide sequences of primer pairs used for quantitative PCR (qPCR) analysis.

**Additional file 15: Table S2**. Antibodies used for immunocytochemical analyses.

**Additional file 16: Table S3**. Median fold-change (FC) mRNA expression of brain cell-type markers. Markers were selected on the basis of extensive literature searches (for the full list of markers examined, see Additional file 11) and cell-specific mRNA expression levels (with a median FC larger than 3.5) in the single-cell human fetal brain RNA-sequencing dataset of Fan et al. (2018) [32]. We noticed that some of the previously reported cell-type-specific markers for the adult brain do not appear to be valid cell-type-specific markers for the fetal brain (Additional file 11). NPC: neural progenitor cell; OPC: oligodendrocyte precursor cell; (p)OL: (pre-myelinating) oligodendrocyte.

## Data Availability

The raw data supporting the conclusions of this article will be made available by the authors upon request. The raw RNA-seq data of batch-1 and batch-12 are available under GEO accession number GSE200779 (encoded “0h” and “0h_2”, respectively).

## Abbreviations

3D: Three-dimensional
ACHE: Acetylcholinesterase
ACTB: Actin Beta
AIF1: Allograft inflammatory factor 1
ALDHL1: Aldehyde Dehydrogenase 1 Family Member L1
ANOVA: Analysis of variance
AQP4: Aquaporin 4
ARG1: Arginase 1
ATP1A2: ATPase Na+/K+ Transporting Subunit Alpha 2
ATP5F1: ATP synthase subunit b
BBB: Blood–brain-barrier
BMP4: Bone morphogenetic protein 4
BSA: Bovine serum albumin
C1QB: Complement C1q B Chain
CCL5: C–C Motif Chemokine Ligand 5
CCL20: C–C Motif Chemokine Ligand 20
CD14: Cluster of differentiation 14
CD143: Angiotensin-converting enzyme
CD80: Cluster of differentiation 80
CD209: Cluster of differentiation 209
CHAT: Choline O-Acetyltransferase
CNP: 2′,3′-Cyclic Nucleotide 3′ Phosphodiesterase
CNS: Central nervous system
COL4A2: Collagen Type IV Alpha 2 Chain
CRTAM: Cytotoxic And Regulatory T Cell Molecule
CX3CR1: C-X3-C Motif Chemokine Receptor 1
CXCL8: C-X-C Motif Chemokine Ligand 8
D: Day
DBH: Dopamine Beta-Hydroxylase
DLX1,2: Distal-Less Homeobox 1
DM: Dorsomorphin
DPBS: Dulbecco's phosphate-buffered saline
ECM: Extracellular matrix
EIF1B: Eukaryotic Translation Initiation Factor 1B
EIF4A2: Eukaryotic Translation Initiation Factor 4A2
ERBB4: Erb-B2 Receptor Tyrosine Kinase 4
(h)ESCs: (Human) Embryonic stem cells
FC: Fold-change
FCGR3A: Fc Gamma Receptor IIIa
FEV: FEV Transcription Factor, ETS Family Member
FOXA2: Forkhead Box A2
FPKM: Fragments Per Kilo base of transcript per Million mapped reads
GABA: γ-Aminobutyric acid
GABRA1: Gamma-Aminobutyric Acid Type A Receptor Subunit Alpha1
GAD1: Glutamate Decarboxylase 1
GAD2: Glutamate Decarboxylase 2
GAPDH: Glyceraldehyde 3-phosphate dehydrogenase
GAT1: GABA transporter 1
GFAP: Glial Fibrillary Acidic Protein
GRIA1: Glutamate Ionotropic Receptor AMPA Type Subunit 1
GRIN1: Glutamate Ionotropic Receptor NMDA Type Subunit 1
GW: Gestational week
hCSs: Human cortical spheroids
HES1: Hes Family BHLH Transcription Factor 1
HLA-DRA: Major Histocompatibility Complex, Class II, DR Alpha
HMGB2: High Mobility Group Box 2
HTR1A: 5-Hydroxytryptamine Receptor 1A
ICC: Immunocytochemistry
IGFBP7: Insulin Like Growth Factor Binding Protein 7
IL-1β: Interleukin-1 beta
IL-6: Interleukin-6
iPSCs: Induced pluripotent stem cells
ITGAM: Integrin Subunit Alpha M
LMX1B: LIM Homeobox Transcription Factor 1 Beta
LPS: Lipopolysaccharide
MAG: Myelin Associated Glycoprotein
MAL: Myelin And Lymphocyte Protein
MAP2: Microtubule-associated protein 2
MBP: Myelin-basic protein
MKI67: Marker of Proliferation Ki-67
MOBP: Myelin Associated Oligodendrocyte Basic Protein
MOG: Myelin Oligodendrocyte Glycoprotein
MS4A1: Membrane Spanning 4-Domains A1
NANOG: Nanog Homeobox
NDRG1: N-Myc Downstream Regulated 1
NEFL: Neurofilament Light Chain
NEUROD6: Neuronal Differentiation 6
NPCs: Neural progenitor cells
OCT4: Octamer-binding transcription factor 4
OLIG1: Oligodendrocyte Transcription Factor 1
OPCs: Oligodendrocyte precursor cells
P2RY12: Purinergic Receptor P2Y12
PAX6: Paired box protein 6
PBS-T: PBS-Tween
PDE4DIP: Phosphodiesterase 4D Interacting Protein
PDGFRA: Platelet Derived Growth Factor Receptor Alpha
PECAM1: Platelet endothelial cell adhesion molecule
PGK1: Phosphoglycerate Kinase 1
Pen-strep: Penicillin–Streptomycin
PFA: Paraformaldehyde
PMP2: Peripheral Myelin Protein 2
PPIA: Peptidylprolyl Isomerase A
PTPRC: Protein Tyrosine Phosphatase Receptor Type C
PVALB: Parvalbumin
qPCR: Quantitative polymerase chain reaction
RAMP3: Receptor Activity Modifying Protein 3
RIN: RNA integrity number
S100B: S100 Calcium Binding Protein B
SALL4: Spalt Like Transcription Factor 4
SATB2: SATB Homeobox 2
SELL: Selectin L
SLC6A3: Solute Carrier Family 6 Member 3
SLC17A7: Solute Carrier Family 17 Member 7
SLCO1C1: Solute Carrier Organic Anion Transporter Family Member 1C1
SMAD: Small Mothers Against Decapentaplegic
SNAP25: Synaptosome Associated Protein 25
SOD2: Superoxide Dismutase 2
SOX9: SRY-Box Transcription Factor 9
SOX10: SRY-Box Transcription Factor 10
SPARC: Secreted Protein Acidic And Cysteine Rich
SPSS: Statistical Package for the Social Sciences
SSM: Spheroid Starter Medium
SYP: Synaptophysin
TEM: Transmission electron microscopy
TGF-β: Transforming growth factor-β
TH: Tyrosine hydroxylase
TMEM119: Transmembrane Protein 119
TNFα: Tumor necrosis factor alpha
TREM2: Triggering Receptor Expressed On Myeloid Cells 2
UGT8: UDP Glycosyltransferase 8
VGLUT1: Vesicular glutamate transporter 1
VIM: Vimentin
VWF: Von Willebrand Factor
YWHAZ: 14-3-3 Protein zeta/delta
