# Supplementary figures and images for: Human cortical spheroids with a high diversity of innately developing brain cell types
Source: Stem Cell Res Ther. 2023 Mar 23;14:50. doi: 10.1186/s13287-023-03261-3 (PMC10035191; doi:10.1186/s13287-023-03261-3)

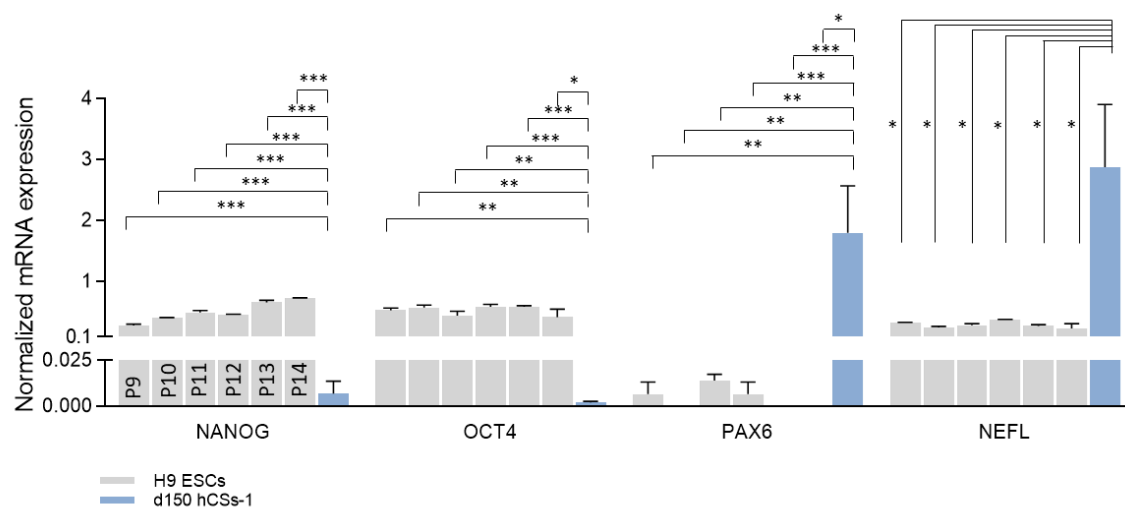

Supplement: Supplementary file 1 — Additional file 1: Fig. S1. The protocol used to culture H9 human embryonic stem cells (ESCs) does not impair their pluripotency. Levels of mRNA expression of stem-cell-specific markers NANOG and OCT4, neural stem cell marker PAX6 and mature neuronal marker NEFL in H9 ESCs (six consecutive passage-numbers, P9-P14) and in day-150 (d150) batch-1 human cortical spheroids (hCSs-1). Error bars represent the standard error of the mean; ANOVA with Bonferroni post hoc comparisons: *** p < 0.001, ** p < 0.01 and * p < 0.05. [file 13287_2023_3261_MOESM1_ESM.pdf]

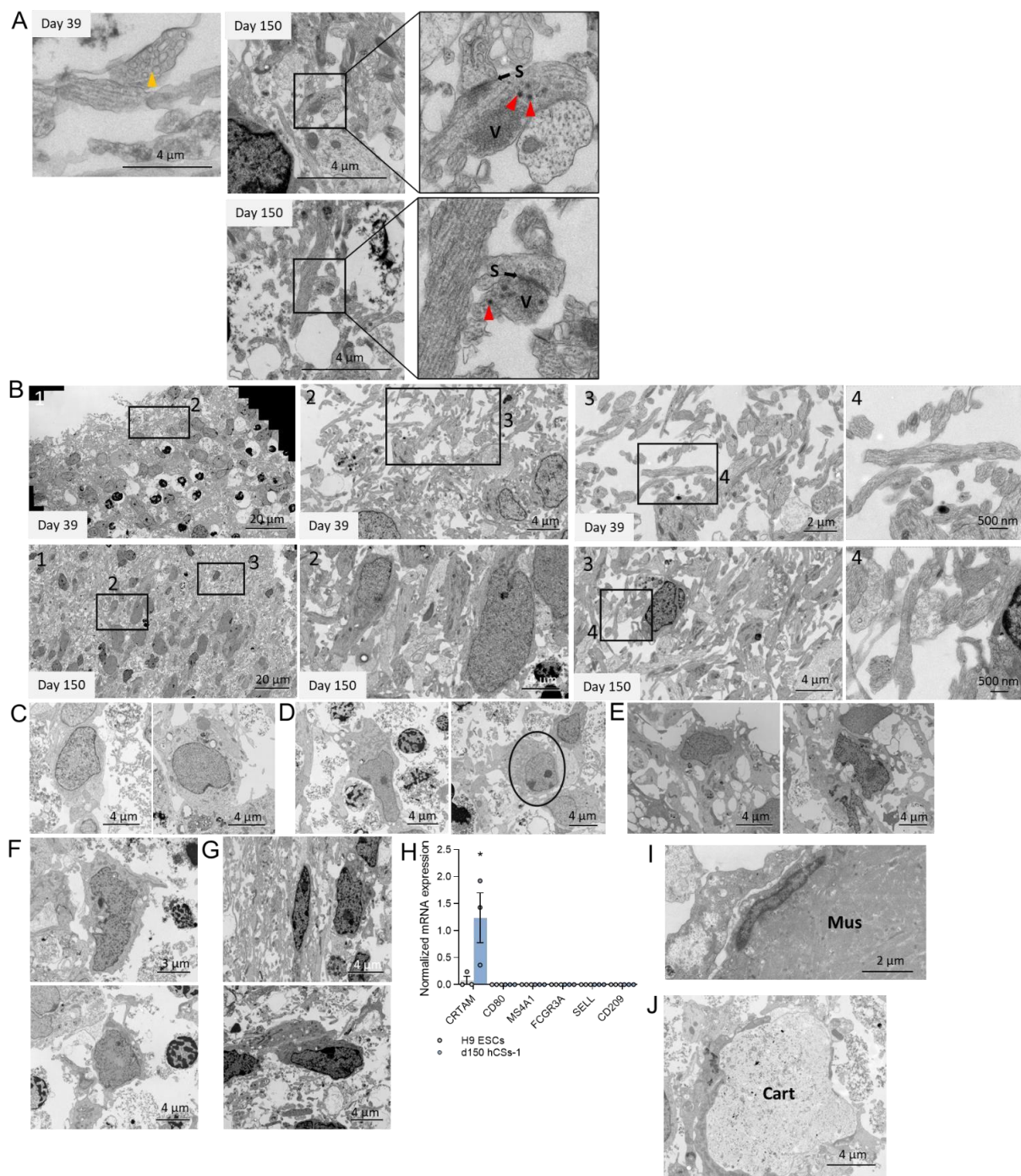

Supplement: Supplementary file 2 — Additional file 2: Fig. S2. Ultrastructural transmission electron microscopy (TEM) analysis of early-stage (day-39, d39) and late-stage (d150) human cortical spheroids (hCSs) reveals multiple neuroectoderm-derived and mesoderm-derived cell types. (A) Left: Synapse formation in d39 hCS (yellow arrow head: synaptic vesicles). Right: Mature synapses (S) with vesicles (V) in d150 hCS (red arrow heads: dense core vesicles). (B) Overview images of d39 and d150 hCSs showing differences in cell density, length of outgrowths and number of cell contacts. Representative images of (C) astrocytes, (D) oligodendrocyte precursor cells (encircled in right image), (E) mature oligodendrocytes, (F) microglia cells and (G) endothelial cells in d150 hCSs. (H) Levels of mRNA expression of the T-cell/neuronal marker CRTAM, the B-cell markers CD80 and MS4A1, the natural killer cell marker FCGR3A, the leukocyte marker SELL and the dendritic cell marker CD209 in H9 human embryonic stem cells (ESCs) and d150 hCSs, as determined by quantitative PCR (qPCR) analysis. Independent samples T-test: * p < 0.05. Error bars represent the standard error of the mean. Each data point represents the level of mRNA expression in one spheroid. (I) Muscle tissue (‘Mus’) observed occasionally in d150 hCS. (J) Cartilage tissue (‘Cart’) observed occasionally in d150 hCS. [file 13287_2023_3261_MOESM2_ESM.pdf]

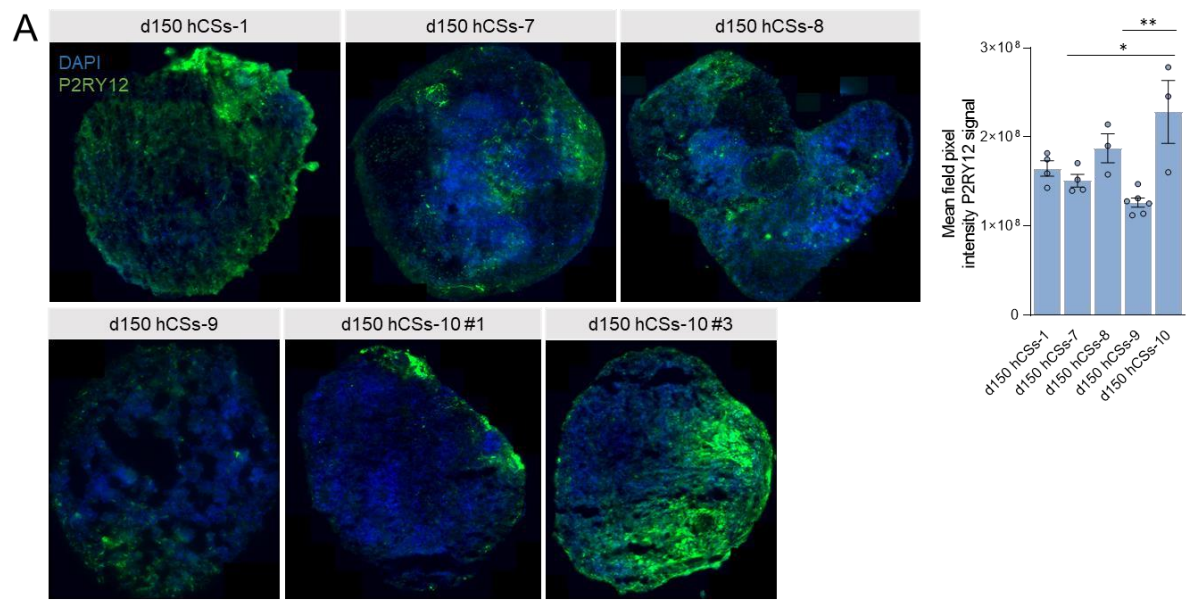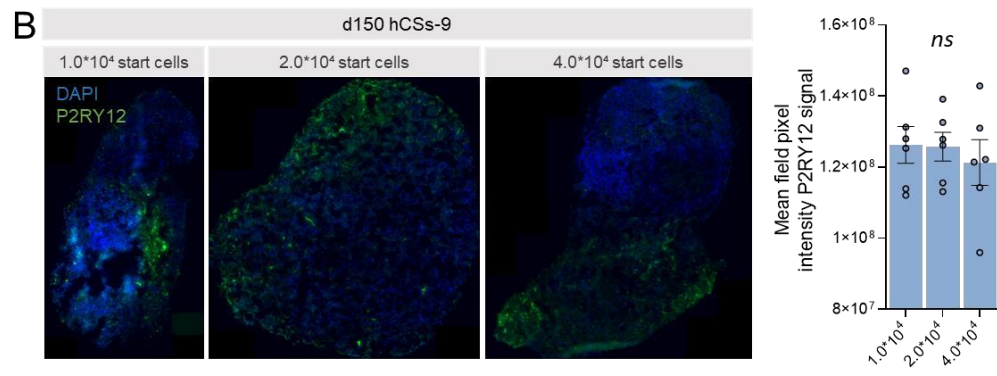

Supplement: Supplementary file 3 — Additional file 3: Fig. S3. Low variability in the number of P2RY12-positive microglial cells among independent human cortical spheroid (hCS) batches and in hCSs grown with various start-cell densities. (A) Left: Representative immunocytochemistry (ICC) images of whole-spheroid signal scans of the microglia marker P2RY12 in five independently grown batches of day-150 (d150) hCSs. Microglia often developed in patch-like structures within the hCSs. Right: Quantifications of the mean P2RY12 signal intensities per field in five independently grown d150 hCS batches. ANOVA with Tukey post hoc comparisons: ** p < 0.01, * p < 0.05. (B) Left: Representative ICC images of whole-spheroid P2RY12 signal scans of d150 hCSs grown with various start H9 human embryonic stem cell (ESC) densities. Right: Quantifications of the mean P2RY12 signal intensities per field in d150 hCSs grown with 1.0*104, 2.0*104 or 4.0*104 start H9 ECSs. ANOVA with Tukey post hoc comparisons: ns, not significant. Each data point in (A) and (B) represents the level of mRNA expression in one spheroid. Error bars represent the standard error of the mean. [file 13287_2023_3261_MOESM3_ESM.pdf]

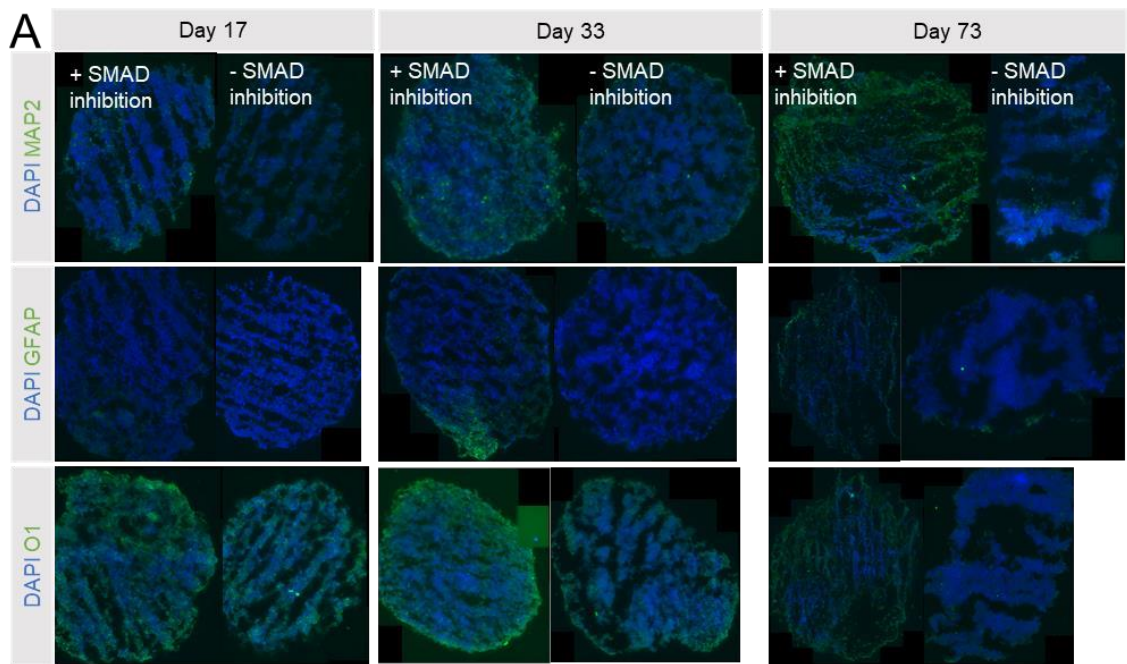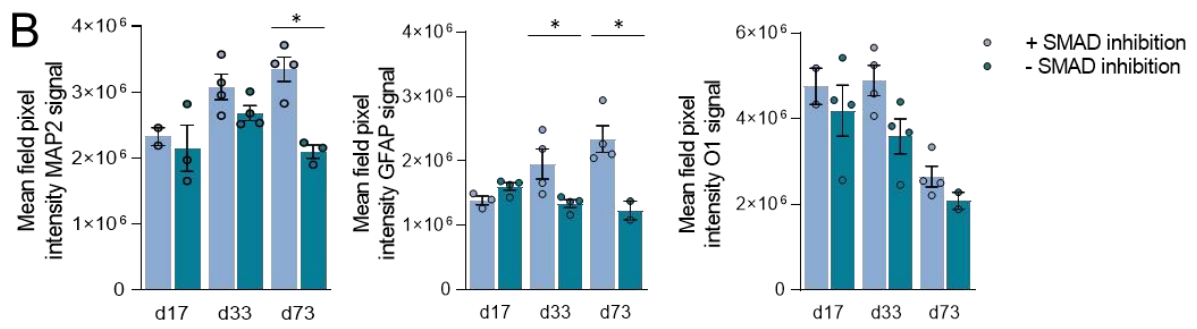

Supplement: Supplementary file 4 — Additional file 4: Fig. S4. Complete omission of SMAD inhibition (absence of dorsomorphin and SB43152 in the culture medium) results in mitigated growth of neuroectoderm-derived neurons and astrocytes. (A) Representative images of whole-spheroid signal scans of the neuronal marker MAP2, the astrocyte marker GFAP and the oligodendrocyte marker O1 in day-17 (d17), d33 and d73 human cortical spheroids (hCSs) (batch-11) grown with dorsomorphin and SB-431542 (+ SMAD inhibition) or without dorsomorphin and SB-431542 (- SMAD inhibition) in the culture medium. (B) Quantifications of the mean MAP2-, GFAP- and O1-signal intensities per field in d17, d33 and d73 hCSs (batch-11) grown with or without SMAD inhibition. One-way ANOVA per time point for -SMAD vs +SMAD: * p < 0.05. Each data point represents the level of mRNA expression in one spheroid. Error bars represent the standard error of the mean. [file 13287_2023_3261_MOESM4_ESM.pdf]

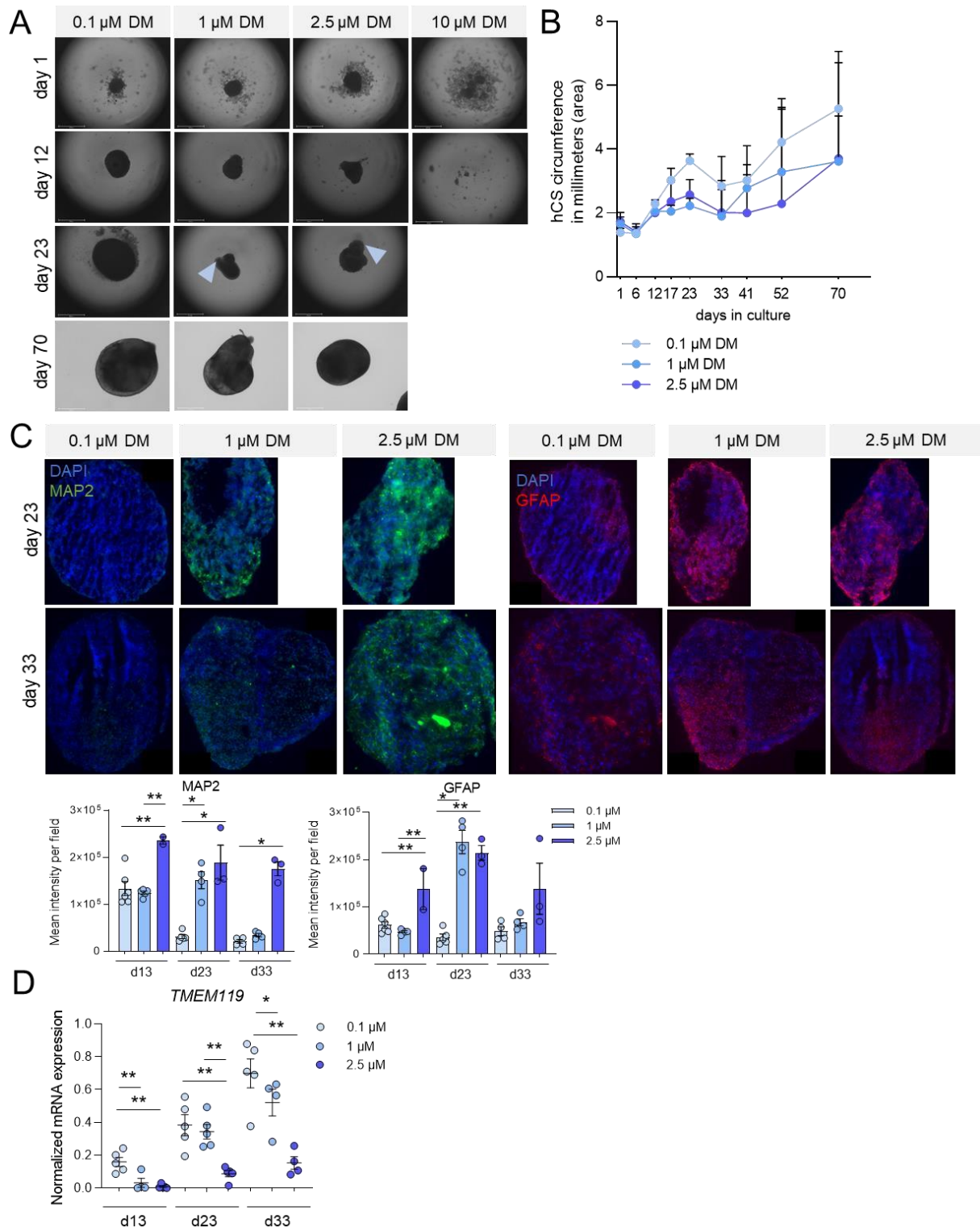

Supplement: Supplementary file 5 — Additional file 5: Fig. S5. Modulation of BMP4 concentration tweaks the ratio between the extent of neuroectoderm- and mesoderm development. (A) Representative images of the morphologies and sizes of human cortical spheroids (hCSs) (batch-2), including neuroectoderm-layer formation/neural rosettes (blue arrow heads) at days 1, 12, 23 and 70 following culturing in the presence of 10 μM SB43152 and 0.1, 1, 2.5 or 10 μM dorsomorphin (DM). hCSs cultured in the presence of a high degree of SMAD inhibition (10 μM DM) and with a starting density of 1.25*104 H9 ESCs did not aggregate into spheres. (B) Quantifications of hCS (batch-2) circumferences during days 0 until 70 following culturing with 10 μM SB43152 and 0.1, 1 or 2.5 μM DM. (C) Representative immunocytochemistry whole-spheroid (batch-2) images and mean field-intensity quantifications of MAP2 (neuron) and GFAP (astrocyte) protein expression in day-13 (d13), d23 and d33 hCSs cultured in the presence of 0.1, 1 or 2.5 μM DM. ANOVA with Tukey post hoc comparisons: ** p < 0.01, * p < 0.05. (D) Levels of mRNA expression of the microglial marker TMEM119 in d13, d23 and d33 hCSs (batch-2) following culturing with 10 μM SB43152 and 0.1, 1 or 2.5 μM DM, as determined by qPCR analysis. ANOVA with Tukey post hoc comparisons: ** p < 0.01, * p < 0.05. Each data point in (C) and (D) represents one spheroid. Error bars represent the standard error of the mean. [file 13287_2023_3261_MOESM5_ESM.pdf]

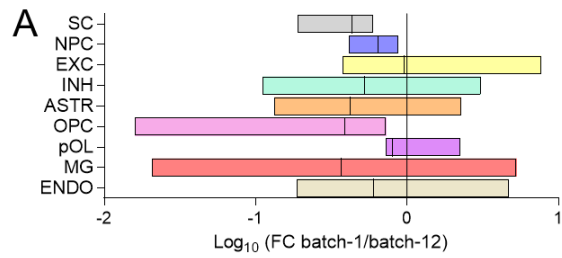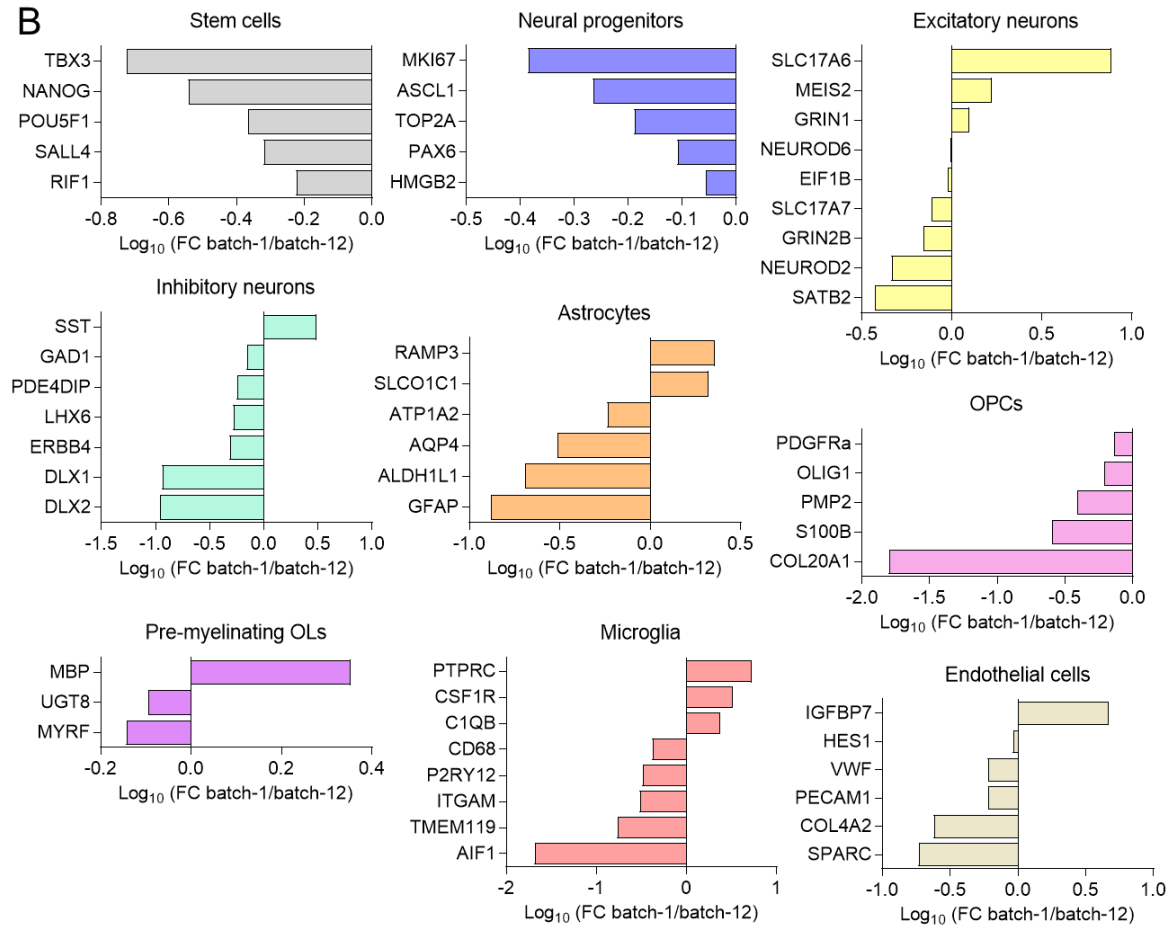

Supplement: Supplementary file 6 — Additional file 6: Fig. S6. Robust presence of both neuroectoderm-derived and mesoderm-derived cell types in two independently grown day-150 (d150) human cortical spheroid (hCS) batches, with slightly different ratios in cell-type composition. (A) Log10-converted fold change (FC) of median GAPDH-normalized mRNA expression of markers for various brain cell types in batch-1 relative to batch-12 d150 hCSs. Each bar represents the median of the expression of all cell-type-specific markers for that brain cell type. (B) Log10-converted FC of median GAPDH-normalized mRNA expression of cell-type-specific markers for various brain cell types in batch-1 relative to batch-12 d150 hCSs. See Additional file 16 for markers used. SC: stem cell; NPC: neural progenitor cell; EXC: excitatory neuron; INH: inhibitory neuron; ASTR: astrocyte; OPC: oligodendrocyte precursor cell; pOL: pre-myelinating oligodendrocyte; MG: microglia; ENDO: endothelial cell. [file 13287_2023_3261_MOESM6_ESM.pdf]

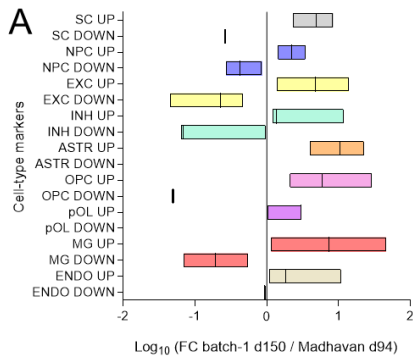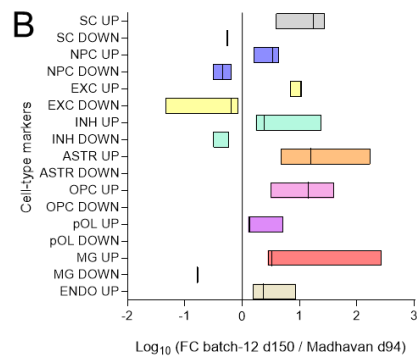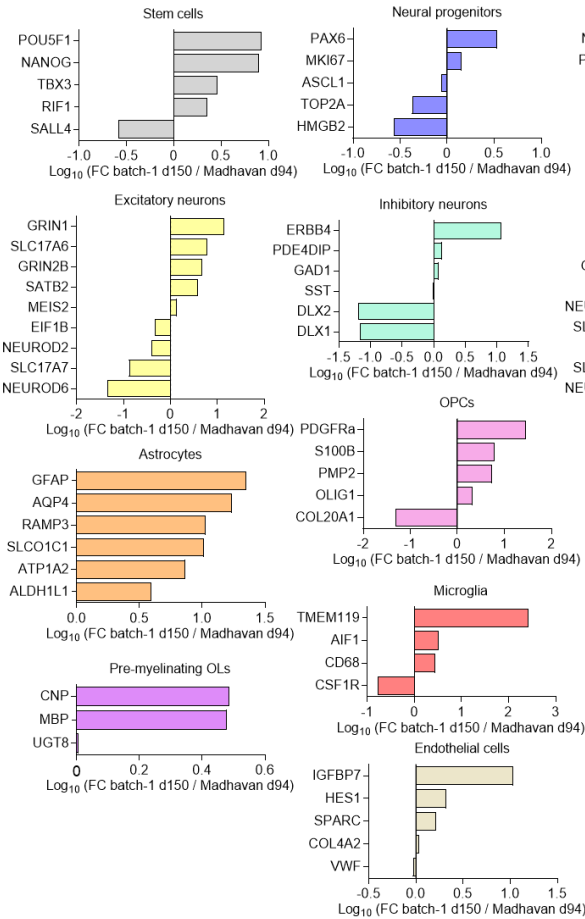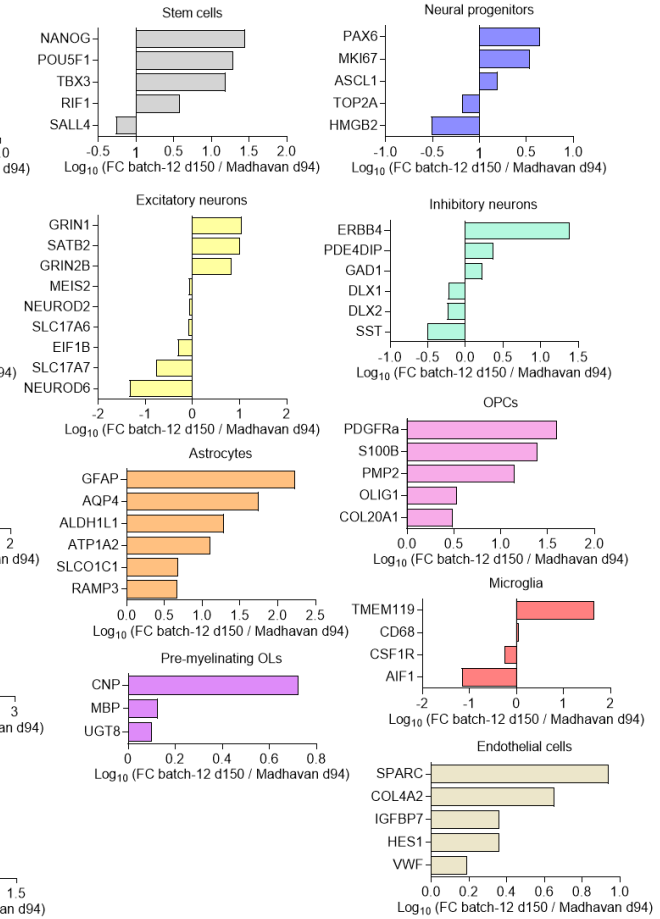

Supplement: Supplementary file 7 — Additional file 7: Fig. S7. Relative distributions of distinct cell types based on transcriptomic comparisons between day-150 (d150) human cortical spheroids (hCSs) generated with our protocol and d94 hCSs generated with the T3-induction protocol of Madhavan et al. (2018). This figure is related to figure 6. (A) Log10-converted fold change (FC) of GAPDH-normalized mRNA expression of cell-type-specific markers in d150 hCSs (batch-1) (present study) relative to that in d94 hCSs (Madhavan et al., 2018). (B) Log10-converted FC of GAPDH-normalized mRNA expression of cell-type-specific markers in d150 hCSs (batch-12) (present study) relative to that in d94 hCSs (Madhavan et al., 2018). [file 13287_2023_3261_MOESM7_ESM.pdf]
