# Supplementary material for: Human cortical spheroids with a high diversity of innately developing brain cell types
Source: Stem Cell Res Ther. 2023 Mar 23;14:50. doi: 10.1186/s13287-023-03261-3 (PMC10035191; doi:10.1186/s13287-023-03261-3)
Supplement: Supplementary file 14 — Additional file 14: Table S1. Nucleotide sequences of primer pairs used for quantitative PCR (qPCR) analysis. [file 13287_2023_3261_MOESM14_ESM.pdf]

Transcript	Forward sequence	Reverse sequence
AIF1	GATCTGCCATCCTAAAAATGATCCT	AGAGATAGCTTTCTTGGCTGGG
AQP4	GGAAATTGGGAAAACCATTGGA	AAGACATACTCATAAAGGCCACCA
ARG1	GACTGGACCCATCTTTCACACC	TCCTGAGAGTAGCCCTGTTTTGT
C1QB	CAGGGATAAAAGGAGAGAAAGGGC	TCTCCCTTCTCTCCGAACTCA
CCL20	GCTGTGACATCAATGCTATCATCT	CTTTTTACTGAGGAGACGCACAA
CCL5	CCATATTCCTCGGACACCACAC	CGAACCCATTTCTTCTCTGGGT
CD14	GGACCTAAAGATAACCGGCACC	TGGGCAATGCTCAGTACCTTG
CD143	CGTGCCTTACATCAGGTACTTTG	TGGACTGGTAGATGTCACACTTGT
CD209	GCTGAGGAGCAGAACTTCCT	GTTGGGCTCTCCTCTGTTCC
CD80	GACCCTAAGCATCTGAAGCCA	TCACGTGGATAACACCTGAACA
COL4A2	CAGGACAGAAAGGAGACCAAGGAG	CCCGATAACCTTTCAGGCCAAATA
CRTAM	TAGGCCAATCTTCCCACCCTA	CTCTGGTACTTGGATGTGCTTT
CX3CR1	CAAATGCCACACATCACCCTTA	TTTGACTCGATGCAGTAGGCAG
CXCL8	GATTTCTGCAGCTCTGTGTGAAGG	GGGTGGAAAGGTTTGGAGTATGT
DLX1,2	GGGACTCACACAGACTCAGGTC	GCTTCATCAGCTTCTTGAACTTGG
EIF4A2	GGTGACATGGACCAGAAGGAGA	CCCCTCTGCCAATTCTGTGAA
FCGR3A	GGATGGGGAAAGGCTGTT	GCCAGCTGAAACTAGAAGTAGC
FGFR3	TCCTGCTCTGGGAGATCTTCAC	TGATCATGTACAGGTCGTGTGTG
GAD1	CCATTCTCGTCAAGGAAAAGGG	ACTGCTTGTCTGGCTGGAAGAG
GAPDH	GTCATGGGTGTGAACCATGAGA	GCATGGACTGTGGTCATGAGTC
GFAP	CAGGACCTGCTCAATGTCAAGC	GTTTCTCGAATCTGCAGGTTGG
GRIN1	GTCCACCAGACTGAAGATTGTGAC	GGTGCAGATCACCTTCTTGACTG
HLA-DRA	GAATTTGGACGATTTGCCAGC	GGAGGTACATTGGTGATCGGAG
IL-6	TGAGGAGACTTGCCTGGTGAA	ACTCATCTGCACAGCTCTGGCT
ITGAM	AGCTTAATACCATCGCATCCAAGC	TGAGTACCCTCGATCGCAAAG
MAG	CCAGGGAGCCCATCGAC	GGTTGTCCCCTGCCGAG
MAL	CTGTACATAATTGGAGCCCACG	ACACCACGGCAGCAATGTT
MAP2	TCGCCATCATACGTACTCCTCC	ATTTGTACCTGCCCCCCTTTAG
MBP	ACCCAAGATGAAAACCCCGTA	GAAATGGCTCGTCACCTTCGT
MKI67	CAGCAACGACATGAAAACCAACA	TGGAGCGCAGGGATATTCCC
MOBP	CCGTTCACCTTCCTCAATTCC	GCTGGTTCTGGTCTTCTGGC
MOG	TTTTGATCCCCACTTTCTGAGG	CGTAGCTCTTCAAGGAATTGCC
MS4A1	TGCTCCAGACCCAAATCTAACA	TTTGAGTTAGCCCAACCACTTC
NANOG	AGATGCCTCACACGGAGACTGTCT	TGGGTTGTTTGCCTTTGGGAC
NDRG1	CTGGAGTCCTTCAACAGTTTGGG	ATAAGGACAAGGCCCTCCACCA
NEFL	GACCCTGGAAATCGAAGCATG	TTGATCGTGTCCTGCATAGCG
NEUROD6	GCCAATCTCTCACAATGCAAGA	TTACTCAGCCCACAAGCATCTG
NPY	CGCTGCGACACTACATCAACC	GGTCTTCAAGCCGAGTTCTGG
OCT4	AGTATCGAGAACCGAGTGAGAGGC	ACCACACTCGGACCACATCCTT
P2RY12	AGGGTCAGATTACAAGAGCACTCA	TATTGCAACCTGCAGAGTGGC
PAX6	CAAATAACCTGCCTATGCAACCC	GCTGACTGTTCATGTGTGTCTGC
PECAM1	ACACGGAAGTTCAAGTGTCCT	GGGAGCCTTCCGTTCTAGAGTA
PLP1	GGCTAGGACATCCCGACAAGTT	ACAGCAGAGCAGGCAAACAC
PPIA	CAGGGTTTATGTGTCAGGGTGG	CCATTTGTGTTGGGTCCAGC
PTPRC	ATGGAGACTATCCTGGAGAACCCT	GCTGTTCATCTAAATTGCAGGATC
SELL	GCCCTCTGTTACACAGCTTCT	TCACAAACTGACACTGGGGC
SLC17A7	GTTGATGAACTGCGGAGGCTTC	GTCCAGGTGGTTCACGTTGAAC
SNAP25	CTTCATCCGCAGGGTAACAAA	TCCATGATCCTGTCGATCTGG
SOD2	TGGTGGAGAACCCAAAGGG	CGTCAGCTTCTCCTTAAACTTGTC
SPARC	TACATCGGGCCTTGCAAATAC	TGTCCTCATCCCTCTCATACAGG

SYP	AGGTGCTGCAATGGGACTTT	GTTGAGTCCCGAGGTCACAG
TGFB2	TCCTGCTAATGTTATTGCCCTC	CTGCACATTTCTAAAGCAATAGGC
TMEM119	CGAGGCACTCTACGGAAACAAG	ACAAAATTTAGCTGGGCGCA
TOP2A	AAGTGTCGTGTCAGACCTTGAAG	TGTAGCAGGAGGGCTTGAAGAC
TREM2	CATCTCCAGGGCTGAGAGACAC	CCAGAGCAGAACAAGGAGTCCT
VWF	CCCTGGGTTACAAGGAAGAAAA	GACCTTGCAGAAGTGAGTATCACA
YWHAZ	CGCTGGTGATGACAAGAAAGG	GAAGTTAAGGGCCAGACCCAGT
