# Supplementary material for: Human cortical spheroids with a high diversity of innately developing brain cell types
Source: Stem Cell Res Ther. 2023 Mar 23;14:50. doi: 10.1186/s13287-023-03261-3 (PMC10035191; doi:10.1186/s13287-023-03261-3)
Supplement: Supplementary file 15 — Additional file 15: Table S2. Antibodies used for immunocytochemical analyses. [file 13287_2023_3261_MOESM15_ESM.pdf]

**Antibody**	**Vendor (catalog#)**	**Host and isotype**	**Dilution**	**Protocol specifications**
CD31 (PECAM1)	NovusBio (JC/70A)	Mouse IgG	1:100	No permeabilization; antibody in PBS-T 0.1%.
GAD2	Sigma (amab91048)	Mouse IgG	1:100	Triton-X in blocking; antibody in PBS-T 0.1%.
GAT1	Sigma (hpa013341)	Rabbit IgG	1:100	Triton-X in blocking; antibody in PBS-T 0.1%.
GFAP	UC Davis/NIH NeuroMab Facility (N206B/9)	Mouse IgG	1:500	Triton-X in blocking; antibody in PBS-T 0.1%.
MAP2	Aves labs	Chicken IgY	1:200	Triton-X in blocking; antibody in PBS-T 0.1%.
O1	Invitrogen (14-6506-82)	Mouse IgM	1:200	Triton-X in blocking; antibody in PBS-T 0.1%.
P2RY12	BioLegend (848002)	Rat IgG	1:250	No permeabilization; antibody in PBS-T 0.1%.
PAX6	Biolegend (PRB278P)	Rabbit IgG	1:100	Triton-X in blocking; antibody in PBS-T 0.1%.
SOX9	Sigma (amab90795)	Mouse IgG	1:100	Triton-X in blocking; antibody in PBS-T 0.1%.
TMEM119	Sigma (HPA051870)	Rabbit IgG	1:500	No permeabilization; antibody in PBS-T 0.1%.
VGLUT1	Abcam (ab227805)	Rabbit IgG	1:500	No permeabilization; antibody in PBS-T 0.1%.
