# Supplementary material for: Human cortical spheroids with a high diversity of innately developing brain cell types
Source: Stem Cell Res Ther. 2023 Mar 23;14:50. doi: 10.1186/s13287-023-03261-3 (PMC10035191; doi:10.1186/s13287-023-03261-3)
Supplement: Supplementary file 16 — Additional file 16: Table S3. Median fold-change (FC) mRNA expression of brain cell-type markers. Markers were selected on the basis of extensive literature searches (for the full list of markers examined, see Additional file 11) and cell-specific mRNA expression levels (with a median FC larger than 3.5) in the single-cell human fetal brain RNA-sequencing dataset of Fan et al. (2018) [32]. We noticed that some of the previously reported cell-type-specific markers for the adult brain do not appear to be valid cell-type-specific markers for the fetal brain (Additional file 11). NPC: neural progenitor cell; OPC: oligodendrocyte precursor cell; (p)OL: (pre-myelinating) oligodendrocyte. [file 13287_2023_3261_MOESM16_ESM.pdf]

Cell type	Marker	Median FC mRNA expression over other CNS cell types	Low discriminative value for (FC < 2.5)
NPC	MKI67	36.95087	Microglia
	TOP2A	27.12461	Microglia
	PAX6	7.046579	Inhibitory neuron, Astrocyte
	HMGB2	15.12611	Microglia
	ASCL1	13.17416	Inhibitory neuron
Excitatory neuron	NEUROD2	6.497525	-
	NEUROD6	7.104337	-
	EIF1B	7.248826	Inhibitory neuron
	MEIS2	7.893909	-
	SATB2	5.694366	-
	SLC17A7	6.639055	-
	GRIN1	5.920607	-
	GRIN2B	62.43829	Inhibitory neuron
	SLC17A6	3.569754	Inhibitory neuron, pOL
Inhibitory neuron	GAD1	12.8172	-
	PDE4DIP	12.7923	-
	DLX1	43.60477	-
	DLX2	36.75871	-
	ERBB4	20.76877	-
	SST	13.65195	-
	LHX6	24.32631	-
Astrocyte	GFAP	6.978993	-
	AQP4	16.65467	-
	SLCO1C1	20.2895	-
	ATP1A2	15.17133	Endothelial cell
	ALDH1L1	16.43695	-
	RAMP3	14.26547	-
OPC	PDGFRa	61.45441	-
	S100B	8.315965	pOL
	OLIG1	15.54875	pOL, NPC
	COL20A1	23.14389	-
	PMP2	6.122841	Astrocyte
pOL	MOBP	285.3296	-
	MYRF	137.7506	-
	MBP	126.2138	-
	UGT8	46.30855	-
	CNP	42.7518	-
Microglia	CD68	41.4727	-
	PTPRC	62.97495	-
	P2RY12	98.51424	-
	C1QB	103.6807	-
	AIF1	92.06095	-
	TMEM119	119.3332	-
	ITGAM	154.5435	-
	CSF1R	48.38399	-
Endothelial cell	SPARC	21.09844	-
	IGFBP7	98.40873	-

	COL4A2	57.49844	-
	HES1	10.70552	-
	PECAM1	23.39329	-
	VWF	37.21952	-
